# Proteomic and ecophysiological responses of soybean (*Glycine max* L.) root nodules to Pb and hg stress

**DOI:** 10.1186/s12870-018-1499-7

**Published:** 2018-11-14

**Authors:** Mohd Affan Baig, Javed Ahmad, Rita Bagheri, Arlene Asthana Ali, Asma Abdulkareem Al-Huqail, Mohamed Mohamed Ibrahim, Mohammad Irfan Qureshi

**Affiliations:** 1 Proteomics and Bioinformatics Lab, Department of Biotechnology, Jamia Millia Islamia (A Central University), New Delhi, 110025 India; 2 Department of Botany and Microbiology, Science College, King Saud University, 11495, Riyadh, Saudi Arabia; 3 Department of Botany and Microbiology, Faculty of Science, Alexandria University, P.O. Box 21511, Alexandria, Egypt

**Keywords:** Soybean, Root nodules, Metal stress, Antioxidants, Proteomics, Real time expression

## Abstract

**Background:**

Lead (Pb) and mercury (Hg) are persistent hazardous metals in industrially polluted soils which can be toxic in low quantities. Metal toxicity can cause changes at cellular and molecular level which should be studied for better understanding of tolerance mechanism in plants. Soybean (*Glycine max* L.) is an important oilseed crop of the world including India. Indian soils growing soybean are often contaminated by Pb and Hg. The aim of this study was to explore how soybean root nodule responds to Pb and Hg through proteomic and ecophysiological alterations in order to enhance tolerance to metal stress.

**Results:**

Soybean plants were exposed to Pb (30 ppm PbCl_2_) and Hg (0.5 ppm HgCl_2_) to study histological, histochemical, biochemical and molecular response of N_2_-fixing symbiotic nodules. Both Pb and Hg treatment increased the level of oxidative stress in leaves and nodules. Chlorosis in leaves and morphological/anatomical changes in nodules were observed. Activities of ascorbate peroxidase, glutathione reductase and catalase were also modulated. Significant changes were observed in abundance of 76 proteins by Pb and Hg. Pb and Hg influenced abundance of 33 proteins (17 up and 16 down) and 43 proteins (33 up and 10 down), respectively. MS/MS ion search identified 55 proteins which were functionally associated with numerous cellular functions. Six crucial proteins namely catalase (CAT), allene oxide synthase (AOS), glutathione S-transferase (GST), calcineurin B like (CBL), calmodulin like (CML) and rapid alkalinisation factor (RAF) were selected for transcript abundance estimation. The qRT-PCR based real time expression exhibited a positive correlation with proteomics expression except for GST and RAF.

**Conclusion:**

Soybean root nodule responds to metal stress by increased abundance of defence, development and repair related proteins. An efficient proteomic modulation might lead to metal-induced stress tolerance in N_2_-fixing nodules. Although concentrations of Pb and Hg used in the study cannot be considered equimolar, yet Hg seems to induce more changes in nodule proteomic profile, and higher damage to both bacteroides and root anatomy.

**Electronic supplementary material:**

The online version of this article (10.1186/s12870-018-1499-7) contains supplementary material, which is available to authorized users.

## Background

Plant roots take up nutrient ions from the soil and while doing so they can pick toxic ions of unwanted elements inadvertently. Such elements include Hg, Pb, Cd, As, etc. which are deleterious for plant health. The US Agency for Toxic Substances and Disease Registry (ATSDR) 2013 ranked such elements in top four priority list of hazardous substances [1].

Soybean is an important oilseed crop due to its high nutritional value. It is a major source of food in terms of protein and oil. Being a member of Leguminosae, soybean is characterised by the presence of nitrogen-fixing nodules in the roots [2]. These nodules home rhizobium for a symbiotic relation which makes soybean far more important apart from source of nutrition. Soybean production improves soil nitrogen content, thus helps in reduced application of nitrogen fertilizer for not only soybean itself but also for subsequent crop on the same soil [3]. Thus, symbiotic nitrogen fixation greatly contributes to sustainable agriculture through its contribution to plant growth, enrichment of nitrogen-deficient soils and limiting the use of nitrogen fertilizers [4].

India is the fifth largest producer (approximately 10.5 million metric tonnes) of soybean in the world. Around half of soybean produce in India comes from Madhya Pradesh [5]. Interestingly, soils of Madhya Pradesh are contaminated with many toxic metals including Hg, As, Pb, etc. [6]. According to Central Pollution Control Board (CPCB), Madhya Pradesh (MP) is accountable for producing more than 85% of hazardous waste which includes high levels of toxic metals including Pb and Hg.

Our understanding for symbiotic interactions has improved well with recent developments in field of multiomics (e.g., transcriptome, proteome and metabolome) and instrumentations (e.g., Real Time PCR and mass spectrometry). There are only few reports on impact of toxic metals on symbiotic nodules [7]. However, there are no reports on proteomic and molecular response of soybean nodules to Pb and Hg. Improving our understanding about the impact of such stress on nodules would help to assess the crop losses, designing strategies for stress management and improvement of stress tolerance against toxic metals.

It has been reported that toxic metals cause reduction in nodulation and nitrogen fixation through decreased cellular water potential, reduced nutrient uptake and induction of oxidative stress [8]. At molecular level, toxic metals can alter expression of key enzymes of metabolic pathways [9]. Metal stress can cause changes in gene expression and protein profile [10]. There are no reports on impact of nodule proteome and real time expression of important genes in soybean under Pb and Hg stress. Therefore, present study was conducted to assess the impact of Pb and Hg on soybean plant with major focus on proteome and transcripts of stress responsive proteins in symbiotic nodules.

## Methods

### Bradyrhizobium japonicum culture

*B. japonicum* strain KAS-1 was obtained from Department of Microbiology, Indian Agricultural Research Institute, Pusa, New Delhi. Bacterial culture was grown at 28 °C for 7 days at 150×g on orbital shaker in yeast extract mannitol broth medium. *Bradyrhizobium japonicum* KAS-1 is a slow growing strain which was identified at IARI, New Delhi. Slow growing rhizobia have better nitrogen fixing efficiency as compared to fast growing rhizobia. The slow growing rhizobia were under log phase at the time of inoculation.

### Plant culture and stress treatment

Soybean (*Glycine max* L. var. JS-335) seeds were obtained from ICAR- Indian Institute of Soybean Research, Indore, Madhya Pradesh (MP), India. Seeds were thoroughly washed with detergent and surface sterilized using 1% sodium hypochlorite for 10 min and rinsed 10 times with autoclaved double distilled water (DDW). Seeds were placed on wet Whatman filter paper in regular petri-dishes and kept overnight in dark for uniform germination. Forty five plastic pots (7″ × 7″; w × d) were divided into three sets viz. control, Lead (Pb) and Mercury (Hg), each pot filled with 1 kg Soilrite® a soil free system having peat, perlite, and vermiculite (1:1:1). Germinated healthy seedlings were transferred to pots. For nodulation, three-days-old seedlings were inoculated with *Bradyrhizobium japonicum* culture. This was done by dispensing 2 mL of bacterial culture (nearly 10^8^ cells mL^− 1^) in a small pit made in Soilrite® in which seedlings were placed by transferring from petri-dish. In soil, plants were watered with nitrogen free nutrient media via wicking system.

**Fig. 1:**
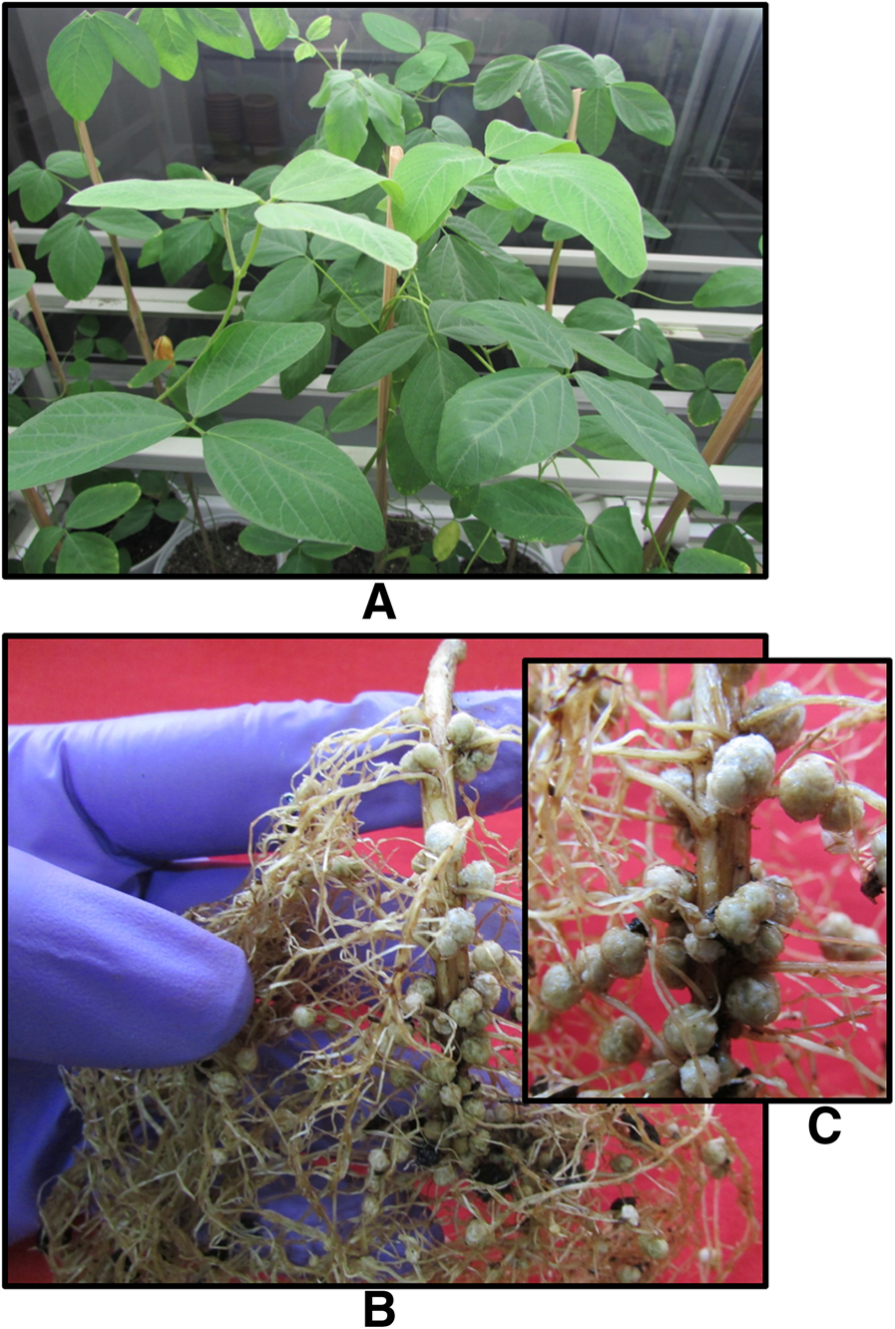
(**a**-**c**). **a**. View of experimental plants at the age of 60 days. **b**. A complete root with mature nodules. **c**. A close up of nodules in the root

For stress induction, 5 pots per set in triplicate were supplied with 30 ppm PbCl_2_ and another set of 5 pots in triplicate were supplied with 0.5 ppm HgCl_2_ dissolved in nutrient solution. One set of 5 pots in triplicates was used as Control, without metal treatment. The concentrations of Pb and Hg were selected as reported in soils of MP. The pots were kept inside a growth chamber under 28 °C temperature and 16 h light of 600 μmol photons m^− 2^ s^− 1^ and 8 h dark cycle. After sixty days (Fig. 1a-c), the plants were carefully up-rooted and the roots were washed with DDW, dried with soft lint-free tissue paper (Kimwipes, Kimberly-Clark, USA). The nodules were harvested from all the sets, immediately frozen in liquid nitrogen, and stored at − 80 °C until used for further study.

### Protein extraction from nodules

Nodule proteins were extracted using ReadyPrep™ total protein extraction kit (Bio-Rad, USA) as per instruction manual. One gram of nodules was pulverized to a fine powder using mortar-pestle in liquid nitrogen. Powder was transferred to Nalgene™ Oak Ridge high-speed centrifuge tubes (Thermo Scientific, USA). 2 mL of 2D rehydration buffer (7 M urea, 2 M thiourea, 1% *w*/*v* amidosulfobetaine-14 detergent, 40 mM tris base and 0.001% bromophenol blue) was added. The extract was centrifuged at maximum speed (15,339×g) for 20 min at 18 °C to pellet bacteria and cell debris. The supernatant was removed and transferred to a clean tube containing cold acetone and kept overnight at − 20 °C for nodule protein precipitation, then centrifuged at 6440×g and supernatant was discarded. Protein pellet was re-suspended in rehydration buffer followed by protein estimation using Bradford reagent [11]. BSA was used as standard.

### 2D (IEF SDS-page)

Following quantification, 200 μg of nodule protein in a total volume of 250 μL in solubilisation buffer was loaded onto 11 cm, non-linear, pH 4–7 IPG (Immobilized pH gradient) strips (Ready Strip™, Bio-Rad, USA) in a rehydration tray. The strips were covered with mineral oil and kept overnight for passive rehydration of proteins. The strips were then placed in isoelectric focusing tray (Protean II IEF™ cell, Bio-Rad, USA) with pre-moistened paper wicks covering each electrode. Protein focusing was performed at 20 °C with total 65,000 Vh current supply in a multi-step programme (Additional file 1: Table S1). After IEF completion, IPG strips having immobilized proteins were equilibrated with reduction buffer (50 mM Tris-HCl, pH 8.8, 8 M urea, 20% (*v/v*) glycerine, 2% (*w*/*v*) SDS and 130 mM DTT) and with alkylation buffer (50 mM Tris-HCl, pH 8.8, 8 M urea, 20% (*v/v*) glycerine, 2% (*w/v*) SDS and 135 mM iodoacetamide) for 20 min each, then rinsed out with ultrapure water. The strips were placed on 12% polyacrylamide gels in a Protean II XL Cell™ (Bio-Rad, USA) for second dimensional run with Precision Plus Protein™ Standards (Bio-Rad, USA) loaded on one side of gels for accurate protein molecular mass determination. Two mL of 0.5% (*w/v*) agarose added with some crystals of bromophenol blue was placed on top of glass plates to serve as a sealing agent and tracking dye, respectively. Electrophoretic run (2nd dimensional) was performed with a current flow of 40 mA per gel at 10 °C. After completion, the gels were stained and de-stained by the method of Candiano at al. [12] for protein visualization. The gels were digitized using gel documentation system (Gel Doc™, Bio-Rad, USA). Protein spot densities were analysed using PDQuest™ software (Bio-Rad, USA). The 2-D gel images of control and stressed plants were thoroughly compared for upregulated and downregulated proteins.

### In-gel tryptic digestion of differentially expressed proteins

The selected protein spots were trypsin-digested according to Bagheri et al. [13]. Spots were picked in 0.2 mL PCR tubes and 100 μL ultrapure water was added, then centrifuged at 101×g, followed by a double wash. To de-stain the gel pieces, 100 μL of 50% (*v/v*) acetonitrile (ACN) was added and tubes were centrifuged at 101×g for 20 min at 22–24 °C. The process was repeated with 100 μL of absolute ACN. 5 μL of 1 M DTT and 45 μL of 100 mM ammonium bicarbonate were added to the tubes and incubated at 56 °C for 45 min. The supernatant was discarded and 100 μL of 0.1 M Iodoacetamide (IAA) in 50 mM ammonium bicarbonate was added to the tubes, then centrifuged at 101×g for 30 min at 22–24 °C. IAA was removed, then 100 μL of 50% (*v/v*) ACN was added to the tubes and centrifuged at 101×g for 20 min at 22–24 °C. ACN was discarded and the step was repeated with absolute ACN. The pellet was dried and incubated for 45 min with 30 μL trypsin (Promega, USA) which was suspended at 1 μg/μL in 50 mM acetic acid and diluted in 50 mM NH_4_HCO_3_ at 20 μg/mL. Trypsin was discarded and 50 μL of 50 mM NH_4_HCO_3_ was added to the tubes and kept overnight (12–16 h) at 37 °C. 5 μL of 1% (*v/v*) TFA was added and centrifuged at 1100×g for 20 min at 22–24 °C. Supernatant was taken in another tube and to the gel slices 100 μL of 50% (*v/v*) ACN and 5% (*v/v*) TFA was added and incubated for 30–60 min at room temperature. Supernatant was added to the first extract. The step was repeated and all the extracts were pooled and freeze dried in a lyophilizer (VirTis, SP Scientific, USA) and stored at − 80 °C until used for further study.

### MALDI-TOF MS/MS analysis for peptide identification

Trypsin digested peptides were dissolved in 0.1% (*v/v*) TFA and mixed with equal volume of matrix (50% *v/v* acetonitrile,0.1% *v/v* TFA and 20 gL^− 1^ α-cyano-4-hydroxycinnamic acid in ultrapure water). The peptide-matrix solution was loaded on to a MALDI plate and allowed to dry. MS/MS analysis was performed on a MALDI-TOF/TOF MS analyzer (AB-SCIEX, TOF/TOF 5800, Applied Biosystems, USA). The peptide mass spectra were detected through result-dependent analysis on Protein Pilot™ v.3.2 software (AB Sciex, MA, USA) with MS (precursor-ion) peak filtering 800–4000 m/z interval, monoisotopic, mass tolerance 50 ppm. MS/MS (fragmentation) peak filtering monoisotopic, MH^+^, minimum signal-to-noise ratio (S/N) 10, MS/MS fragment tolerance 0.75 Da. Protein identification was done using MASCOT database search engine (Matrix Science, London, UK) with MS/MS ion search performed against non-redundant NCBI protein database. Parameters were set as taxonomy-viridiplantae, enzyme-trypsin with one missed cleavage, fixed modification to carbamidomethyl C, peptide and MS/MS tolerance set to 1 Da and 0.8 Da respectively with 2+ peptide charge. Significant hits with MASCOT probability-based score (*p* < 0.05) and best matched molecular weight, *pI* value and percent sequence coverage were considered to evaluate protein identification.

### qRT-PCR based gene expression analysis

Six proteins from among those involved in stress response were selected for further confirmation at transcriptional level through qRT-PCR expression analysis. The genes which were selected on the basis of proteomic results of soybean nodules are catalase (CAT), allene oxide synthase (AOS), gluitathione S-Transferase (GST), calcineurin B like (CBL), calmodulin like (CML) and rapid alkalinisation factor (RAF). Actin (ACTIN) was used as the standard. The gene sequences were retrieved from NCBI nucleotide database. For every gene, the corresponding forward and reverse primers were designed (Additional file 2: Table S2) using primer3Plus software (http://www.bioinformatics.nl/cgi-bin/primer3plus/primer3plus.cgi). Total nodule RNA from control and stressed plants was isolated using RNeasy plant mini kit (Qiagen, USA). RNA was quantified using BioPhotometer plus (Eppendorf, Germany). One μg of total RNA was reverse transcribed using M-MuLV RT-PCR kit (Merck, Germany). qRT-PCR was done with three replicates on Rotor Gene™ 6000 real time rotary analyzer (Corbett Research, Australia) using SYBR green master mix, gene specific primers and cDNA in a final volume of 20 μL with actin as internal control. For data normalisation CT values of genes were subtracted from CT value of reference gene (ΔCT) and relative quantification (2^-ΔΔCT^) was calculated by subtracting ΔCT of interest from ΔCT of reference gene. Information about assay and run conditions has been presented in (Additional file 3: Table S3, Additional file 4: Table S4).

### Nodule oxidative damage and antioxidant enzyme assays

Thiobarbituric acid reactive substances (TBARS) was estimated to measure magnitude of oxidative stress by the method of Heath and Packer [14]. 0.5 g fresh nodules were grounded to a fine powder using liquid nitrogen in a mortar and pestle. The powder was homogenized in 1% (*w/w*) TCA (10 mL g^− 1^ FW) and centrifuged for 5 min at 10062×g. To the fresh tube 1.0 mL supernatant and 4.0 mL of 0.5% (*w/v*) TBA were added. The homogenate was incubated at 95 °C for 30 min, then immediately cooled on ice bath and centrifuged at 4200×g for 5 min to form a clear solution. The absorbance of supernatant was read at 532 nm and the value was subtracted from absorbance value at 600 nm for correction of unspecific turbidity.

For measurement of antioxidant enzyme activities fresh nodules from each treatment were harvested, washed with distilled water, and blot dried. Enzyme extract was prepared by homogenizing 0.5 g nodules in a pre-chilled mortar and pestle with 100 mM potassium phosphate buffer (pH 7.5) containing 3 mM DL-dithiothreitol, 1 mM EDTA, 5% (*w/v*) insoluble polyvinyl pyrrolidone and 1 Mm ascorbic acid. The solution was centrifuged at 11,269×g for 30 min and supernatant was kept at -20 °C until enzyme assays were performed. Ascorbate peroxidase (APX) assay was performed by measuring the absorbance of supernatant at 290 nm according to Qureshi et al. [15]. A unit of enzyme is defined as the amount of APX required to oxidize 1 μmol of ascorbate per min at 25 °C (2.8 mmol^− 1^ cm^− 1^extinction coefficient of ascorbate at 290 nm). The activity of catalase (CAT) was measured by the decrease in absorbance of supernatant at 240 nm due to degradation of H_2_O_2_ as reported by Aebi et al. [16]. A unit of enzyme is defined as the amount of catalase required to decompose 1 μmol of H_2_O_2_ per min with molar extinction coefficient of 0.04 mmol^− 1^ cm^− 1^ at 240 nm absorbance. Glutathione reductase (GR) activity was performed by the method of Anderson [17].The decrease in absorbance of reaction mixture at 340 nm was recorded to access the enzyme activity. A unit of enzyme is defined as the amount of GR that catalyzes the reduction of 1 μmol of GSSG min^− 1^ mg^− 1^ protein.

### Histochemical assays for detection of hydrogen peroxide and superoxide in leaf

The accumulation of hydrogen peroxide (H_2_O_2_) and superoxide (O_2˙ˉ_) anion in leaves was histochemically assessed by 3,3′-diaminobenzidine (DAB) and nitroblue tetrazolium (NBT) staining of soybean leaves following the procedure described by Scarpeci et al. [18].

#### Visualization of hydrogen peroxide

For detecting H_2_O_2_, the leaves were immersed overnight in a solution of DAB (1 mg mL^− 1^, pH 3.8). Thereafter, chlorophyll was removed by boiling in ethanol for 10 min and the reddish-brown coloration denoting the H_2_O_2_ content was visualized and photographed.

#### Visualization of superoxides

For detection of O_2˙ˉ_, the leaves were floated in 50 mM sodium phosphate (pH 7.5) containing 0.2% NBT and the dark blue insoluble formazan compound formed by reaction of NBT with O_2˙ˉ_ which was visualized in leaf and photographed.

### Nodule growth related parameters

Immediately after harvesting plants were washed with DDW and blot-dried. Plants were dissected into nodules, roots, stems and leaves. Nodules were counted and expressed in per plant basis. Each organ was carefully weighed, and fresh weight (FW) was recorded as FW per plant in grams. Material was placed in hot-air oven at 65 °C for a week. Dry weight was recorded as DW per plant in grams.

### Study of nodule transverse section by light microscopy

Fresh nodules from control and stressed plants were cut into 1–3 mm^2^ pieces and kept in solution for fixation containing 2.5% (*w/v*) glutaraldehyde, 2% (*v/v*) paraformaldehyde and 1% (*v/v*) formaldehyde. The fixing solution containing nodule pieces was vacuum infiltrated for 10 min and stored at 4 °C for overnight. The sections were washed with 0.1 M phosphate buffer (pH 7.4) and kept for 2 h in osmium tetraoxide solution at 4 °C followed by washing with 0.1 M phosphate buffer (pH 7.4). The samples were added with 30–90% (*v/v*) acetone for dehydration and placed in dry acetone (saturated with copper sulphate) at 4 °C for 1 h. Samples were then treated twice with toluene for 1 h each and kept overnight in resin and toluene (1:3) under vacuum, then impregnated in resin and toluene (2:2 and 3:1) overnight in vacuum. The samples were finally impregnated in pure resin at room temperature for 6 h. Samples were cut into 500 nm sections and stained in 1% methylene blue for 40s. The sections were observed with bright field microscope (CX31, Olympus, UK).

### GO term enrichment and statistical analysis

Gene ontology enrichment analysis for protein identification was carried out using agriGO database (http://bioinfo.cau.edu.cn/agriGO/) [19]. Protein spot intensities were log2 transformed for hierarchical clustering and partial least squares discriminant analysis (PLS-DA) using Metaboanalyst 3.0 software [20]*.* All data are presented as the mean ± standard error (SE). The measurements were made using five biological replicates. The statistical analyses were carried out using one-way analysis of variance (ANOVA) and means were compared using Tukey’s test. NS = Non-significant, *P ≤ 0.05 and **P ≤ 0.01.

## Results

### Pb and hg induced oxidative stress in leaves

**Fig. 2:**
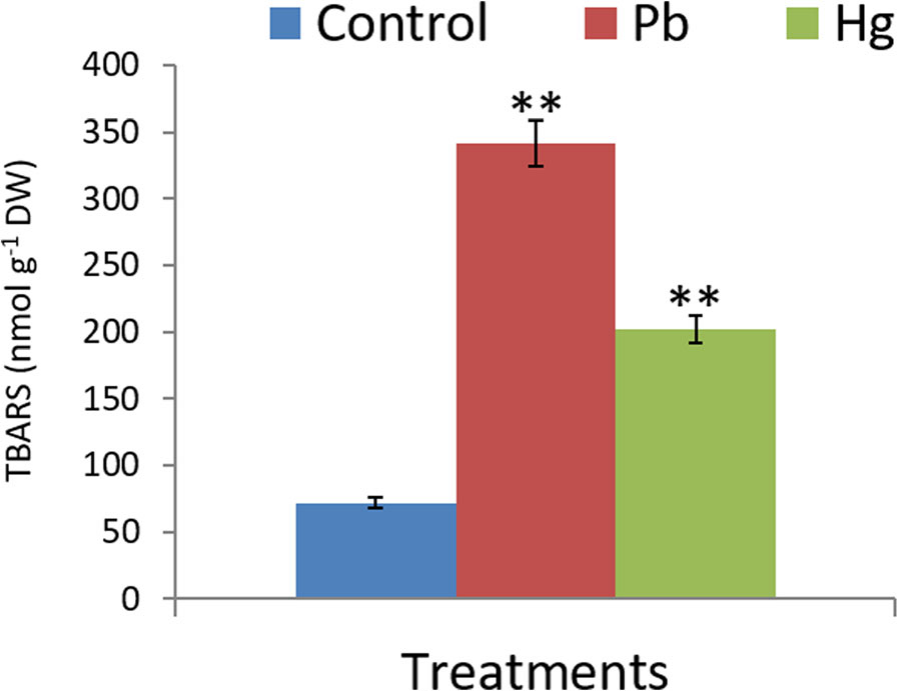
Impact of Pb and Hg on magnitude of oxidative stress in *Glycine max*l. Leaves. Oxidative stress marker was estimated in terms of TBARS content. All data are presented as the mean ± standard error (SE). The measurements were made using five biological replicates. The statistical analyses were carried out using one-way analysis of variance (ANOVA) and means were compare using Tukey’s test. NS = Non-significant, *P ≤ 0.05 and **P ≤ 0.01

Both Pb and Hg induced oxidative stress in leaves. Control, Pb and Hg treated plants accumulated 72, 342 (~ 4 fold) and 202 (~ 2 fold) nmol TBARS g^− 1^ DW, respectively (Fig. 2).

### Histochemical assays demonstrated oxidative stress in leaves

#### Histochemical detection of hydrogen peroxide

**Fig. 3:**
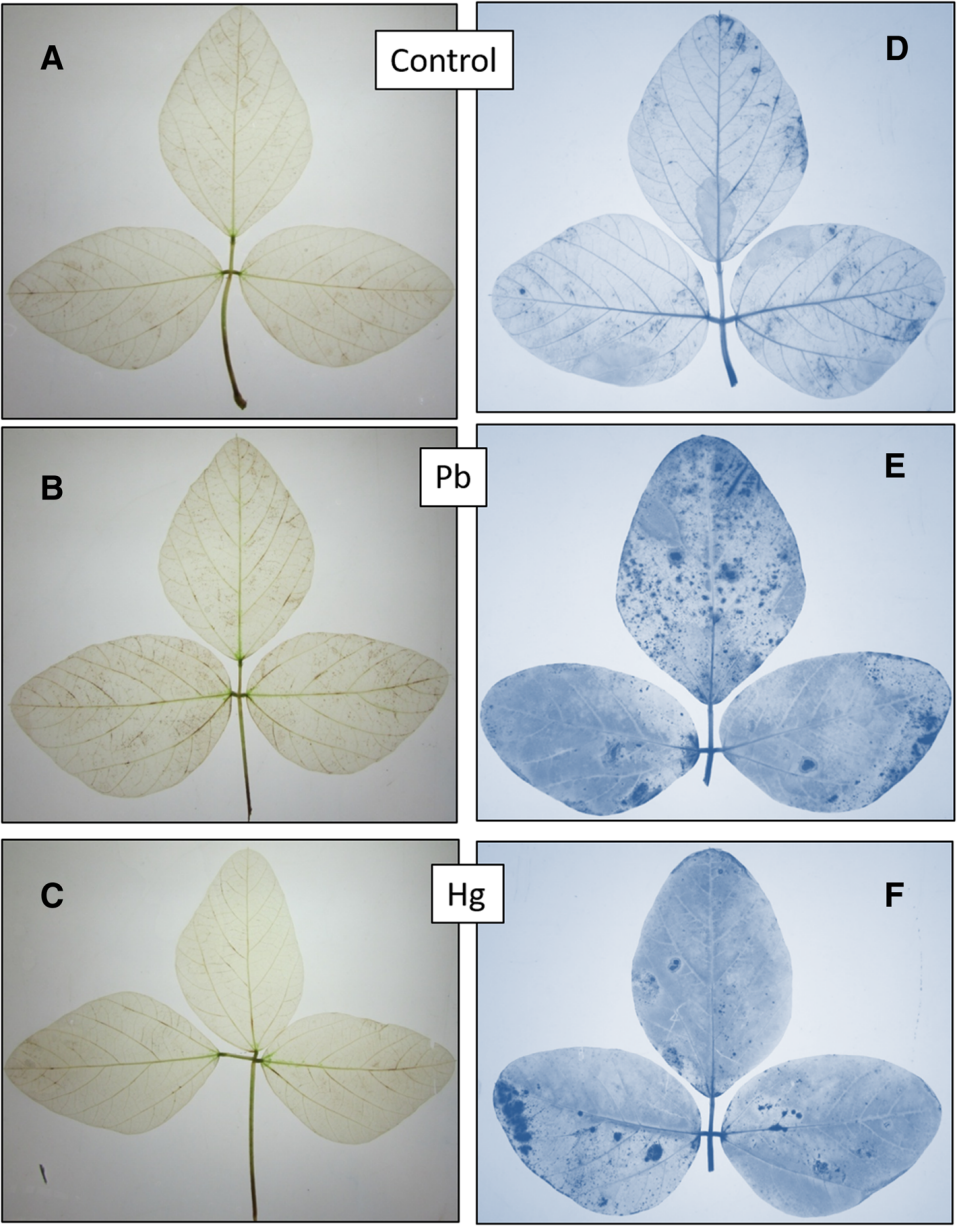
(**a-f**). *Glycine max* L. leaves showing localization of H_2_O_2_under control (**a**), Pb (**b**) and Hg (**c**) treatments and superoxidesunder control (**d**), Pb (**e**) and Hg (**f**) treatments

Results show that Pb increased the concentration of hydrogen peroxide in the leaves. However, Hg did not make much difference compared to control (Fig. 3a-c).

#### Histochemical detection of superoxides

Images show that both Pb and Hg elevated the levels of superoxides as compared to control (Fig. 3d-f).

### Impact of Pb and hg on growth parameters and nodule anatomy

Soybean growth and yield attributes were significantly affected by Pb and Hg treatment after 60 days of treatment (DAS). Pb and Hg resulted into a decline of nodule number by 31 and 60%, nodule fresh weight by 17 and 47%, nodule dry weight by 6 and 30%, respectively. Treatment of Pb and Hg resulted into a decline of fresh weight and dry weight of root, stem and leaf. Pb and Hg treatment caused decline of root fresh weight by 19 and 43%, root dry weight by 25 and 47%, stem fresh weight by 20 and 34%, stem dry weight by 22 and 39%, leaf fresh weight by 24 and 38%, leaf dry weight by 24 and 42% respectively (Table 1).

**Table 1:** Effect of lead (Pb) and mercury (Hg) on nodule number, fresh weight (FW) and dry weight (DW) of nodule, root, stem and leaf in Soybean (*Glycine max* L.) at 60 DAS

Attributes per plant	Treatment		
	Control	Lead (Pb)	Mercury (Hg)
Nodules			
Number	138 ± 13.3 (00)	95 ± 7.67 (−31.16%)*	58 ± 4.67 (−57.97%)**
Fresh weight (g)	1.327 ± 0.06 (00)	1.101 ± 0.05 (−17.03%) ^NS^	0.704 ± 0.04 (−46.95%)**
Dry weight (g)	0.405 ± 0.03 (00)	0.380 ± 0.01 (−6.17%) ^NS^	0.250 ± 0.01 (−38.27%)**
Root			
Fresh weight (g)	5.97 ± 0.21 (00)	4.85 ± 0.30 (−18.8%) ^NS^	3.37 ± 0.18 (−43.55%)**
Dry weight (g)	0.588 ± 0.03 (00)	0.44 ± 0.03 (−25.17%)*	0.31 ± 0.02 (−47.27%)**
Stem			
Fresh weight (g)	7.13 ± 0.42 (00)	5.72 ± 0.38 (−19.77%) ^NS^	4.73 ± 0.25 (−33.66%)***
Dry weight (g)	1.29 ± 0.06 (00)	1.00 ± 0.08 (−22.48%)*	0.78 ± 0.06 (−39.53%)**
Leaf			
Fresh weight (g)	9.77 ± 0.85 (00)	7.37 ± 0.65 (−24.56%)*	6.02 ± 0.42 (−38.38%)**
Dry weight (g)	1.56 ± 0.08 (00)	1.20 ± 0.04 (−23.8%)*	0.91 ± 0.05 (− 41.67%)**

All data are presented as the mean ± standard error (SE). The measurements were made using five biological replicates. The statistical analyses were carried out using one-way analysis of variance (ANOVA) and means were compare using Tukey’s test. ^NS^ = Non-significant, *P ≤ 0.05 and **P ≤ 0.01

**Fig. 4:**
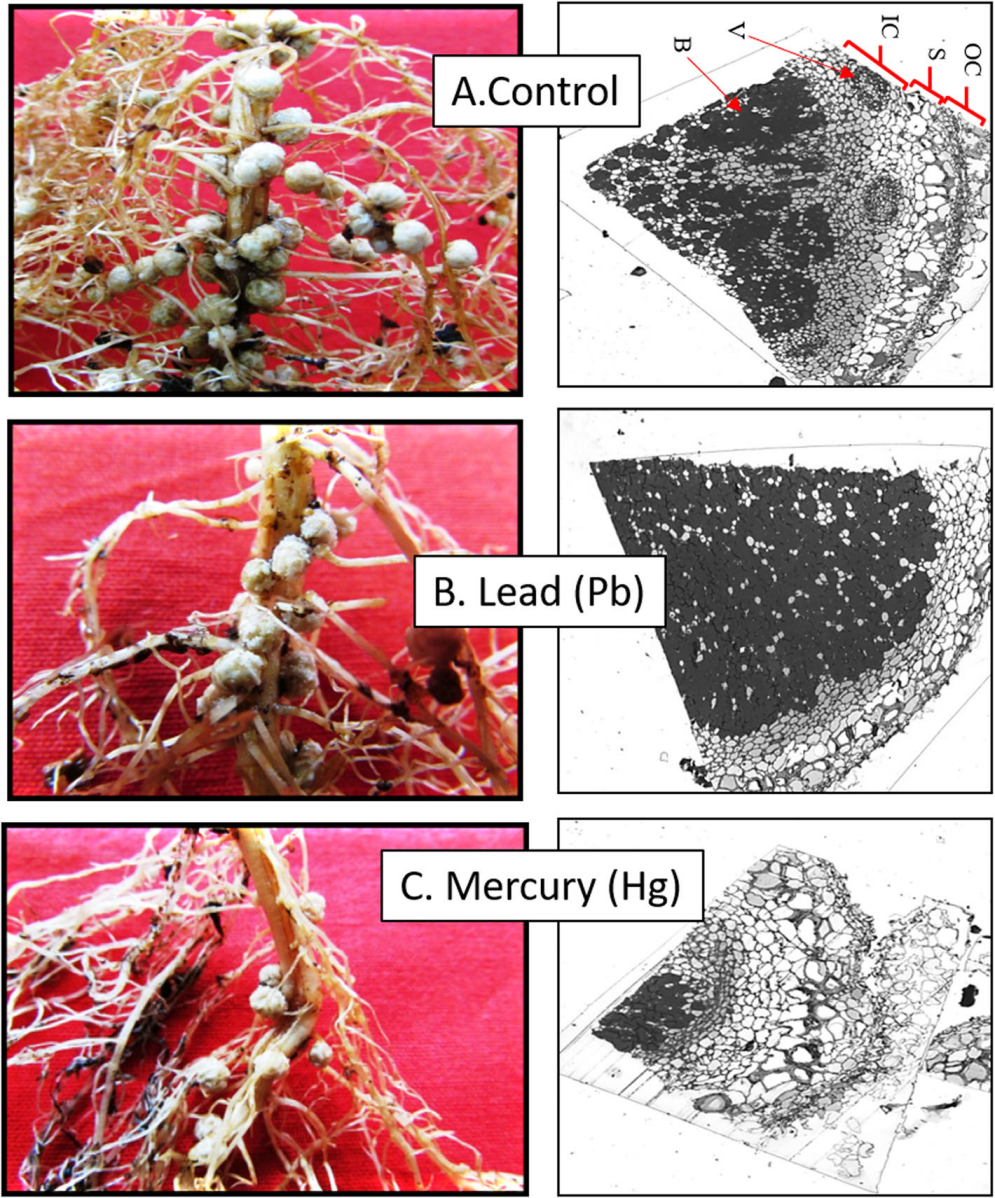
**a-c**. Impact of Pb (30 ppm), and Hg (0.5 ppm) on morphology and anatomy of Soybean roots and nodules. Nodule components are OC = outer cortex, S = sclerid layer, IC = inner cortex, V = vascular bundle, B = bacteroids

Nodule transverse section (TS) of control plants showed all the components of root vasculature besides well-formed bacteroides (Fig. 4a). However, root fibres were lesser, and population of bacterial cell was dense in nodules of Pb treated plants with non-visibility of regular vasculature (Fig. 4b). Nodule TS of Hg treated plants showed overall damage to both vasculature and bacteroides (Fig. 4c).

### Pb- and hg-induced oxidative stress in root nodules

**Fig. 5:**
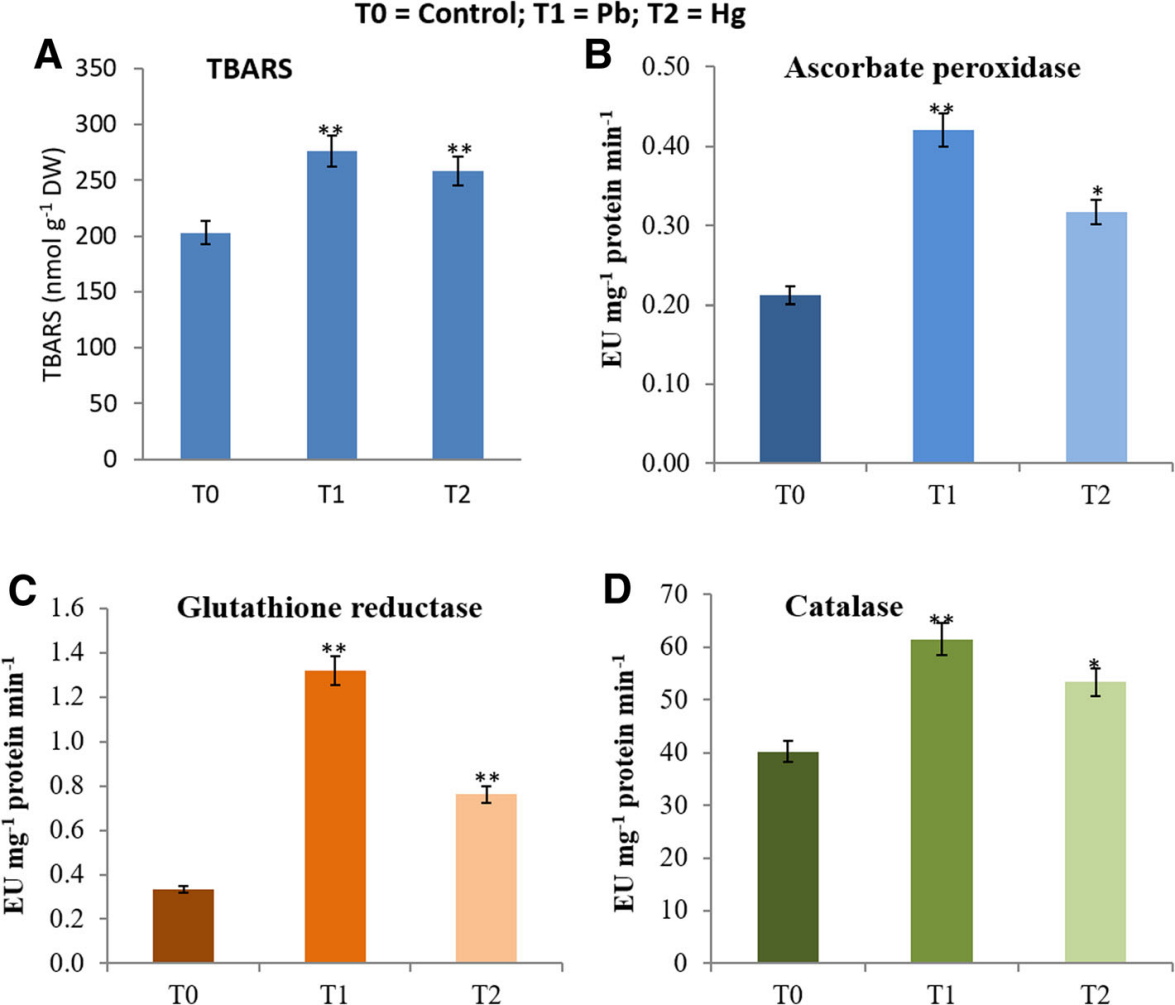
**a-d**. Impact of Pb and Hg on magnitude of oxidative stress (**a**) and changes in activities of ascorbate peroxidase (APX, **b**), catalase (CAT, **c**) and glutathione reductase (GR, **d**). Enzyme actities have been expressed in enzyme units (EU) mg^− 1^ protein min^− 1^ in soybean root nodules exposed to Pb (30 ppm) and Hg (0.5 ppm). All data are presented as the mean ± standard error (SE). The measurements were made using five biological replicates. The statistical analyses were carried out using one-way analysis of variance (ANOVA) and means were compare using Tukey’s test. NS = Non-significant, *P ≤ 0.05 and **P ≤ 0.01

Nodules of Control, Pb and Hg treated plants accumulated 203, 276 (+36%) and 258 (+27%) nmol TBARS g^− 1^ DW, respectively (Fig. 5a).

### Pb and hg induced changes in antioxidant enzymes

Activity of enzymes of ascorbate-glutathione antioxidant system viz. ascorbate peroxidise (APX) and glutathione reductase (GR) got modulated in response to Pb as well as Hg (Fig.5b-d). Control, Pb- treated and Hg-treated plants showed 0.212 EU, 0.421 EU (+98%) and 0.317 EU (+49%) respectively, of APX activity (Fig. 5b). GR activity in control, Pb-treated and Hg-treated plants was 0.333 EU, 1.32 EU (+296%) and 0.762 EU (+128%), respectively (Fig. 5c).

Activities of another important antioxidant enzyme, catalase (CAT), were found to be around 40 EU (Control), 61 EU (Pb-treated) (+53%) and 53 EU (Hg-treated) (+33%) (Fig. 5d).

### Impact of Pb and hg stress on relative abundance of nodule proteins

Untargeted comparative proteomics for differential abundance of proteins, to explore response mechanism of nodule to Pb and Hg, was taken into account.

### Differential relative expression of proteins on 2D gels

**Fig. 6:**
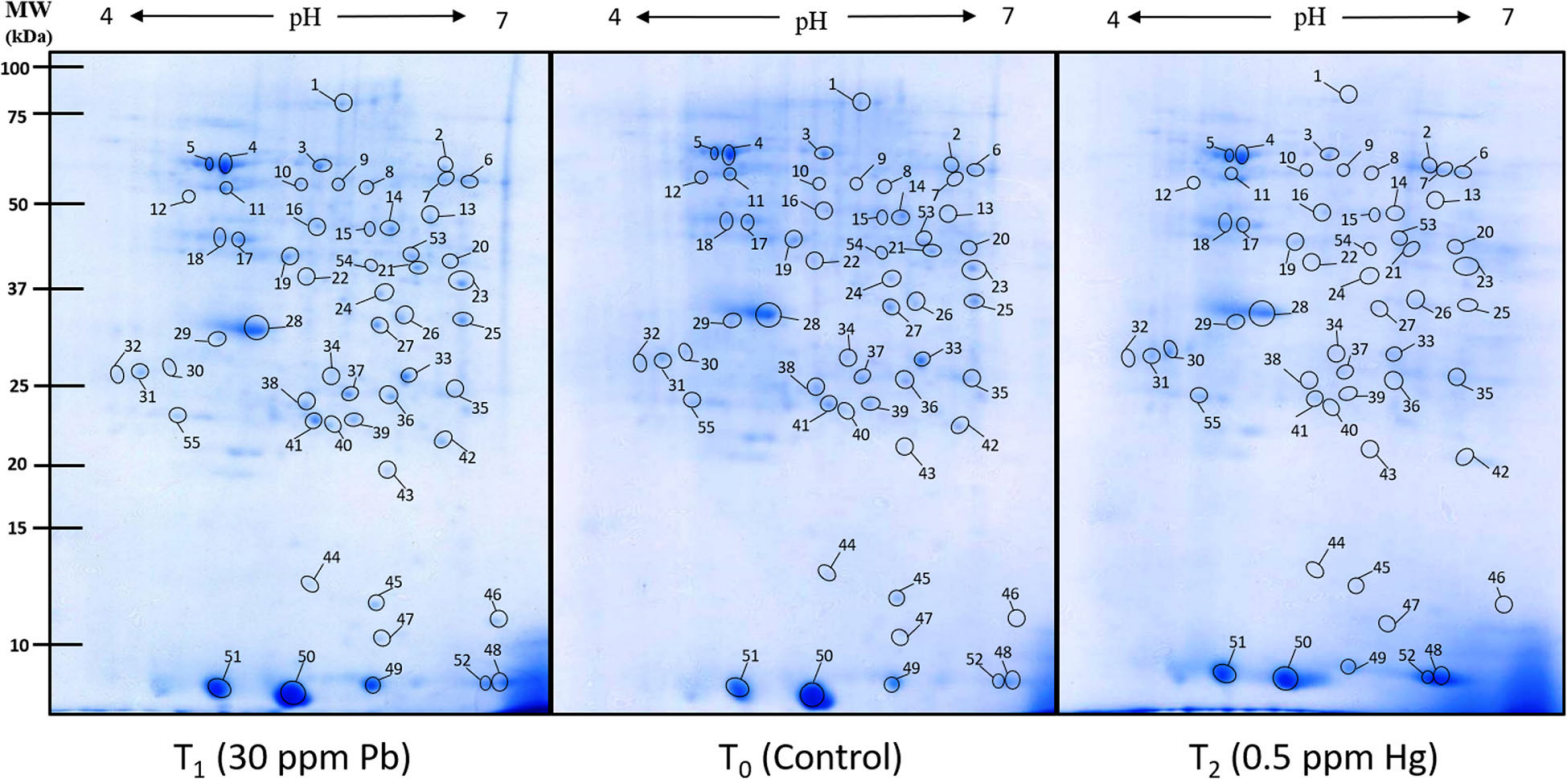
Representative 2D proteome maps of soybean root nodules of control, Pb-treated (30 ppm) and Hg-treated (0.5 ppm). 400 μg of total protein was loaded on to 11 cm/non-linear/pH 4–7 IPG strips for first dimensional run on IEF cell followed by second dimensional run on 12% SDS-PAGE. The proteins were visualised by staining of gels with blue silver stain. The encircled protein spots with numbering represents differentially expressed proteins which were considered for tryptic digestion and subsequent identification through MALDI-TOF MS/MS

**Fig. 7:**
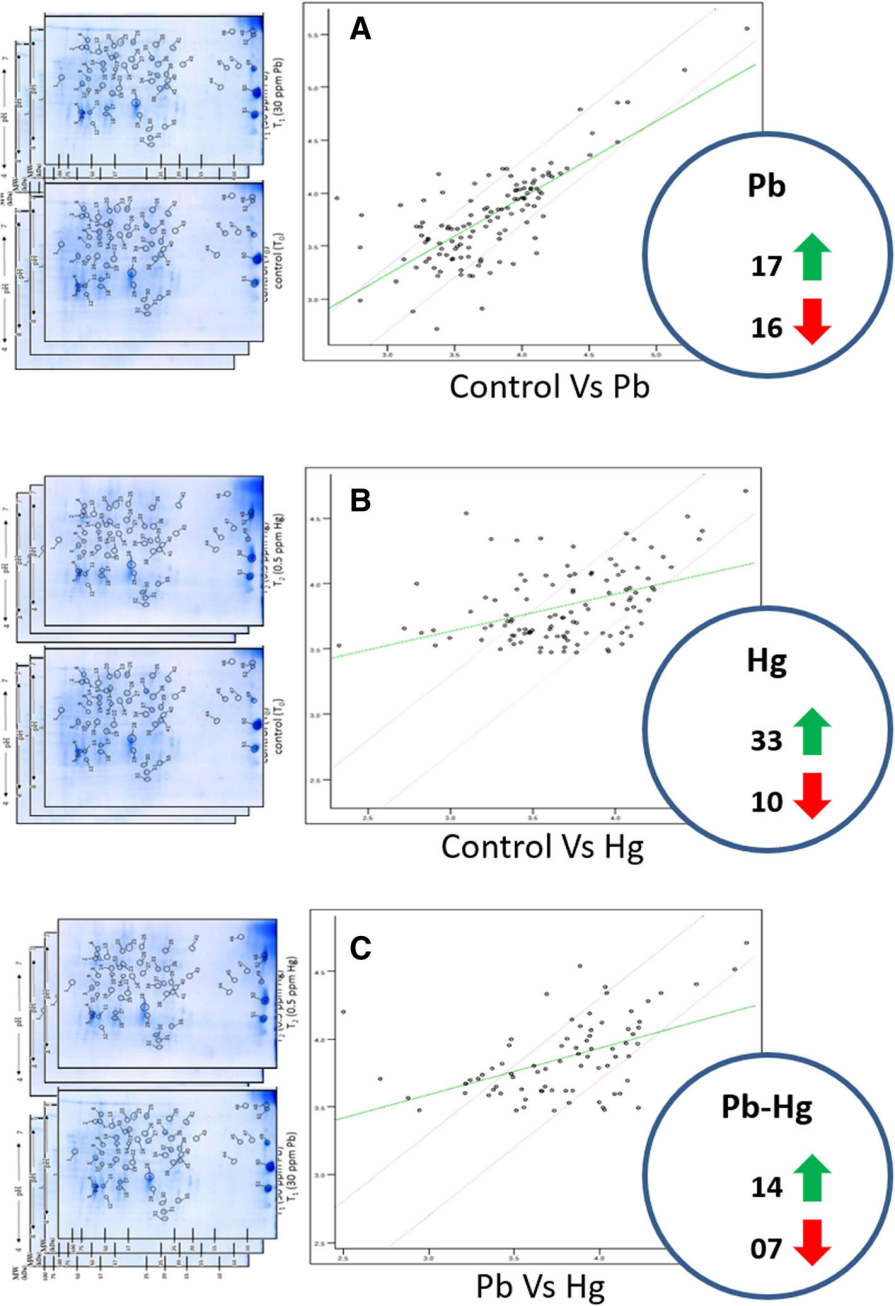
(**a-c**). Image analyses of 2D proteome maps for computing the changes in expression of protein profile as affected by Pb and Hg in root nodules of *Glycine max*

Expression of altered proteome was successfully visualised on 2D gels (Fig. 6) and digitized for comparative analysis using PDQuest™ (Bio-Rad, USA). Around 245 spots were reproducibly detected on each gel. Comparative image analysis showed that overall 76 spots were two-fold differentially expressed against control, out of which 33 were expressed in Pb treatment (17 upregulated and 16 downregulated) and 43 spots in Hg treatment (33 upregulated and 10 downregulated) (Fig. 7).

### Hierarchical clustering and partial least squares discriminant analysis

**Fig. 8:**
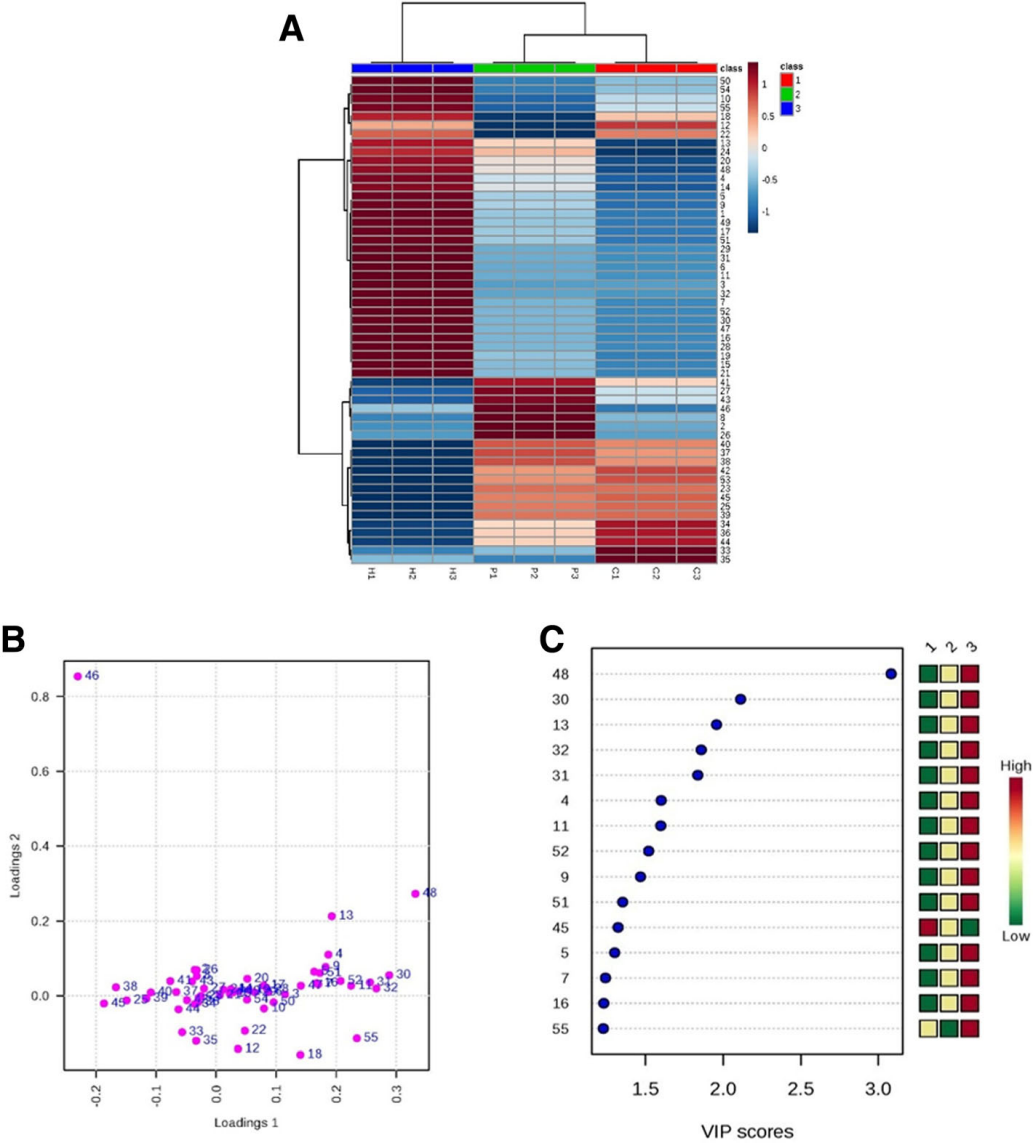
(**a-c**). Multivariate data analysis of differentially expressed proteins in root nodules of Soybean performed by MetaboAnalyst software. Hierarchical clustering (**a**), Loadings plot (**b**) and VIP plot (**c**) displays differentially expressed proteins with maximum abundance in Control (1)_,_Pb (2) and Hg (3) treatments

Hierarchical clustering was performed for analyzing correlation between protein profiles and changes between Control, Pb and Hg treatments. Protein spot intensities were normalized by log transformation for analysis by MetaboAnalyst software (Fig. 8a-c). Hierarchical clustering showed variation between differentially expressed proteins (Fig. 8a). Variable importance in projection (VIP) plot was created from PLS-DA loading plots (Fig. 8b) for identification of differentially expressed proteins with maximum abundance (Fig. 8c). Fifteen protein spots were identified with VIP score (1.5–3) which include ring finger protein (spot 55), mitochondrial transcription termination factor protein (spot 16), probable L-gulonolactone oxidase 6 (spot 7), aluminium activated malate transporter (spot 5), probable calcium binding protein CML43 (spot 45), V-ATPase related protein (spot 51), hydroperoxide lyase (spot 9), glutaredoxin C-5 like protein (spot 52), armadillo/beta-catenin repeat family protein (spot 11), catalase 3 (spot 4), MADS box transcription factor (spot 31), calcineurin B-like protein (spot 32), allene oxide synthase (spot 13), SCF ubiquitin ligase (spot 30), and bet1-like SNARE protein (spot 48).

Proteins with significant changes in relative abundance and belonging to a variety of stress response (Additional file 5: Figure S1) were selected to study the quantitative real time expression by targeting at transcriptional level.

### Identification and functional categorization of nodule proteins

**Fig. 9:**
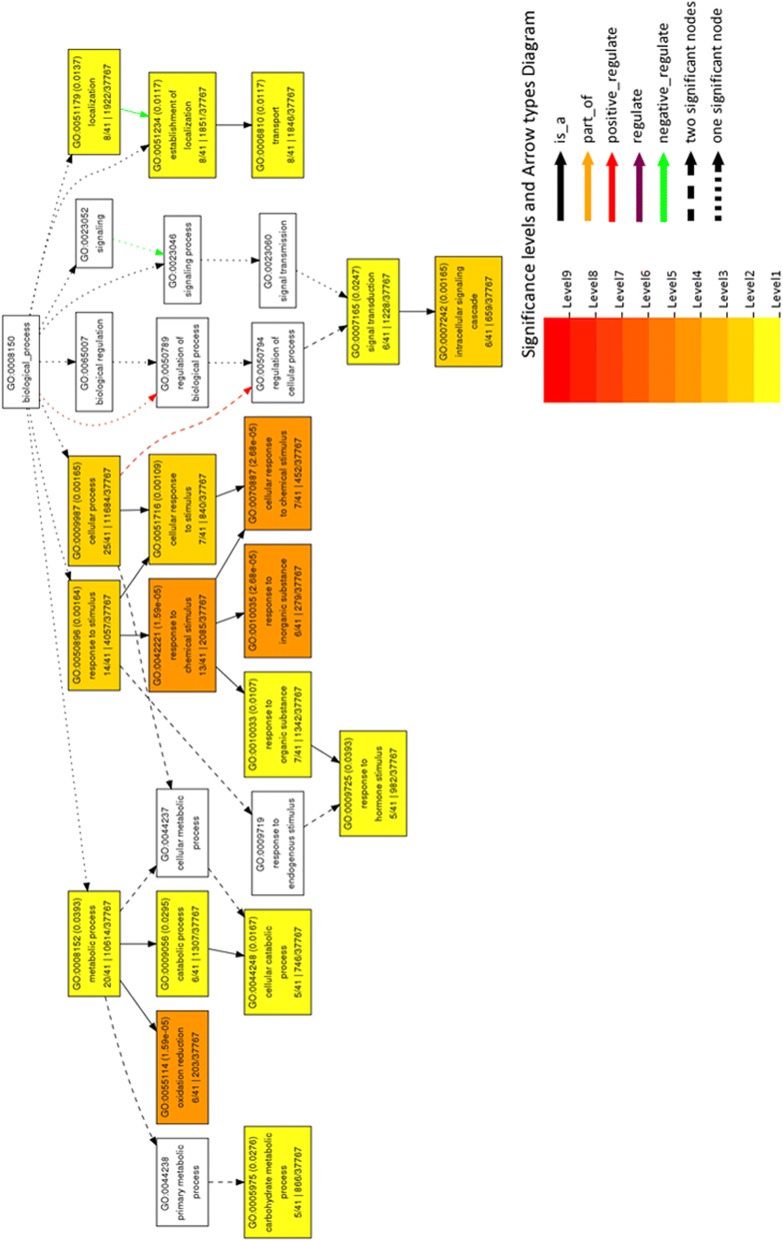
GO term enrichment analysis of differentially expressed proteins using AgriGo database corresponding to biological function

**Fig. 10:**
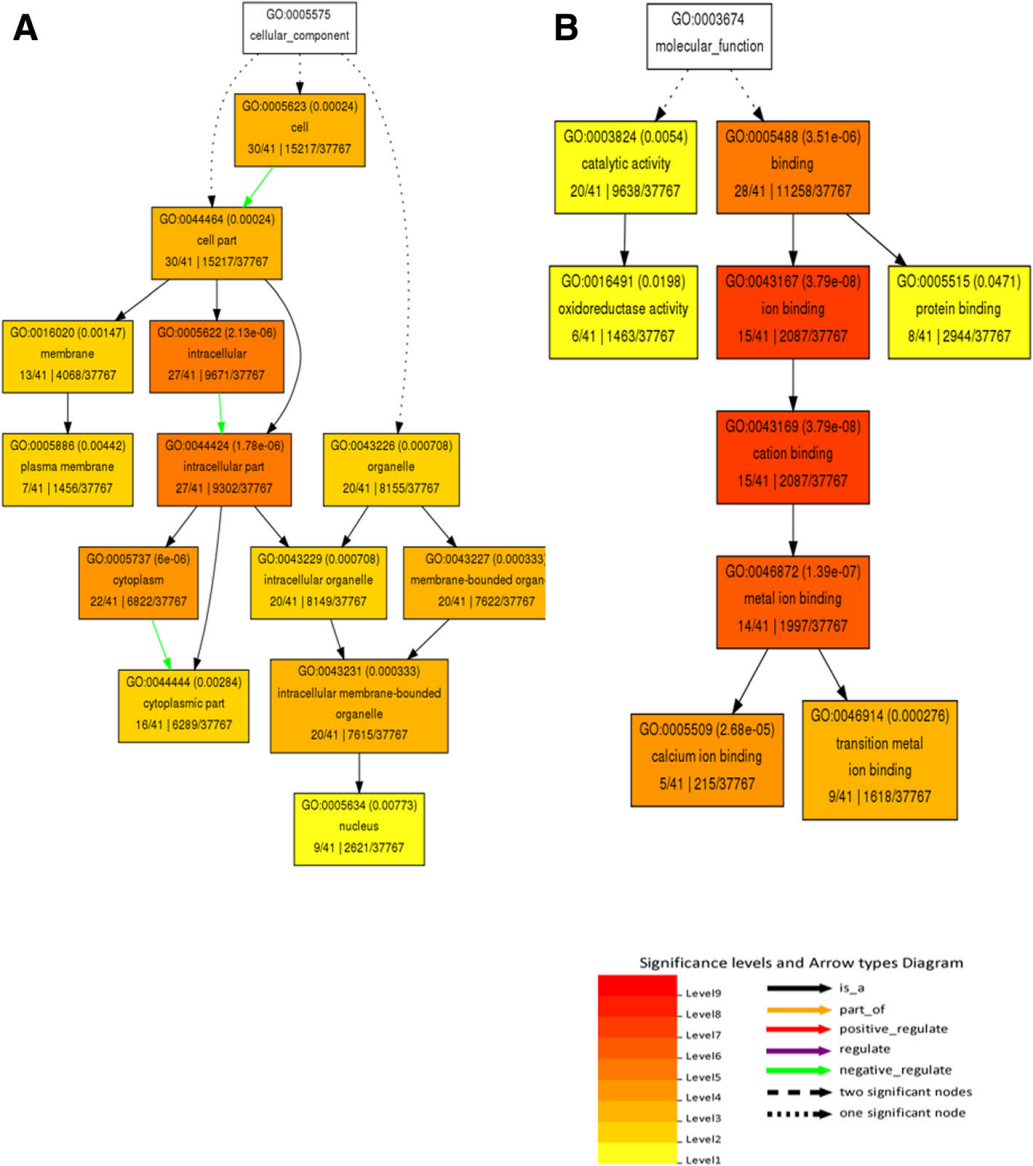
(**a-b**). GO term enrichment analysis of differentially expressed proteins using AgriGo database corresponding to (**a**). Cellular components and (**b**). Molecular function

Fifty-five differentially expressed protein spots were selected for tryptic digestion and identification using MALDI TOF MS/MS followed by MS/MS ion search on MASCOT database (http://www.matrixscience.com/). The identified proteins were searched against UniProt database (http://www.uniprot.org/) for their biological function and cellular component. Homologous soybean gene ID’s were retrieved from SoyKB database (http://soykb.org/). Gene ontology enrichment analysis with agriGO database shows functional distribution of proteins (Fig. 9 and Fig. 10a-b). Biological process shows an overall increase in metabolic process, hormone signalling and signal transduction (Fig. 9) Cellular component shows decrease in membrane and organelle activity (Fig. 10a) while molecular function has increased oxidoreductase activity and protein binding with decreased calcium and metal ion binding (Fig. 10b).

Additional file 6: Table S5 describes various parameters of identified proteins including theoretical/practical molecular weight and *pI* values, number of peptides matched, per cent sequence coverage, NCBI accession, SoyKB gene ID’s, biological function, and cellular location.

**Fig. 11:**
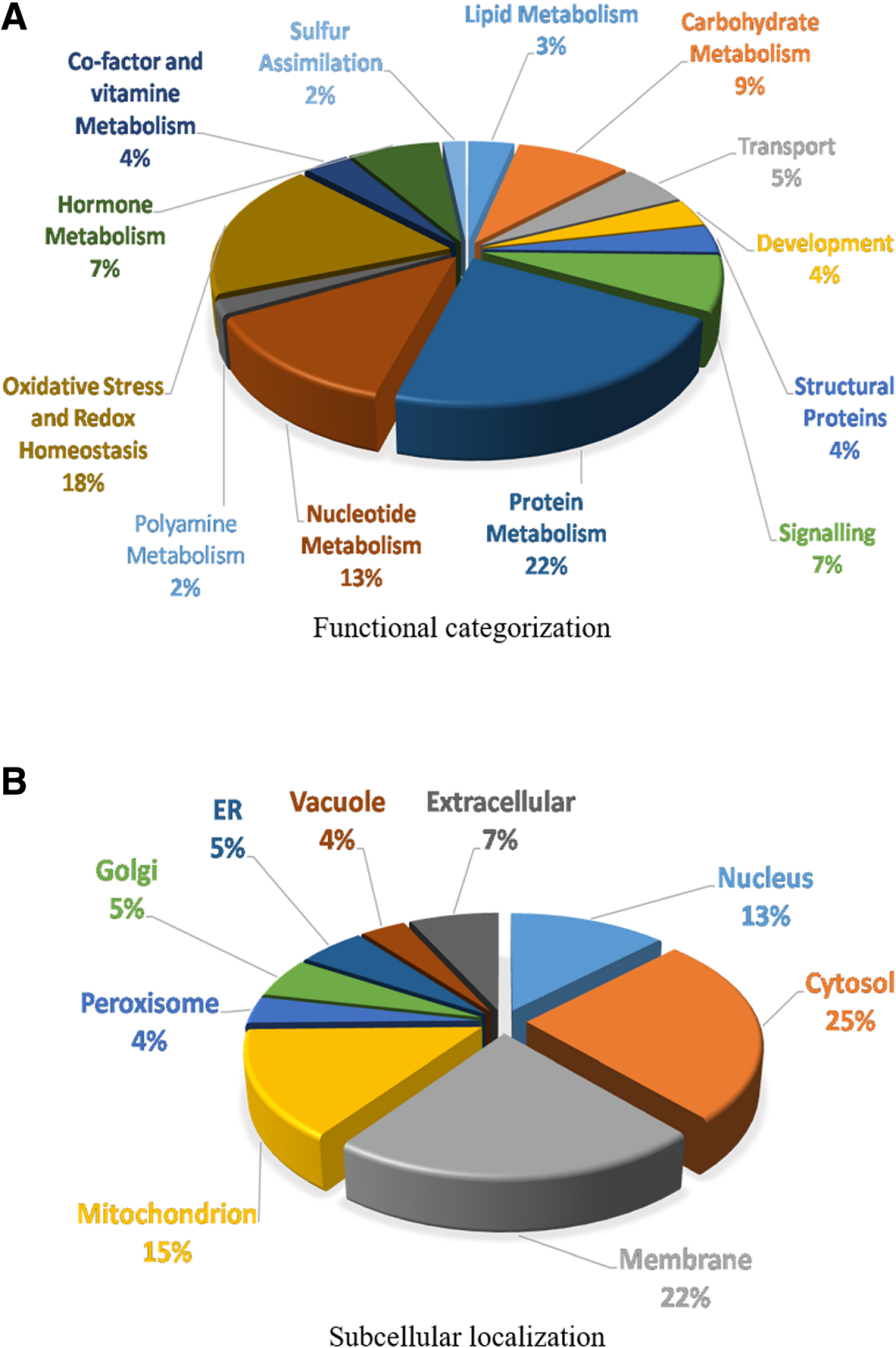
**a-b**. Functional categorization (**a**) and subcellular localization (**b**) of differentially expressed proteins in soybean root nodules exposed to Pb and Hg

The identified proteins were found associated with diverse biological functions (Fig. 11a) with maximum contribution to protein metabolism (22%), oxidative stress response (18%) and nucleotide metabolism (13%) which might be due to Pb and Hg toxicity leading to activation of defence, cell division and repair process. Proteins with differential expression involved in various other vital cellular functions were also identified such as carbohydrate metabolism (9%), signalling (7%), hormone metabolism (7%), transport (5%), development (4%), structural proteins (4%), co-factor and vitamin metabolism (4%), lipid metabolism (3%), sulfur assimilation (2%) and polyamine metabolism (2%).

### Categorization of differentially expressed proteins at sub-cellular level

The proteins were further categorized based on their cellular location (Fig. 11b) with maximum proteins located in cytosol (25%), membrane (22%), mitochondrion (15%) and nucleus (13%). Proteins located in other cell locations are less abundant such as extracellular proteins (7%), endoplasmic reticulum (5%), Golgi (5%), vacuole (4%) and peroxisomes (4%).

### Comparative study of gene transcripts with protein expression

**Fig. 12:**
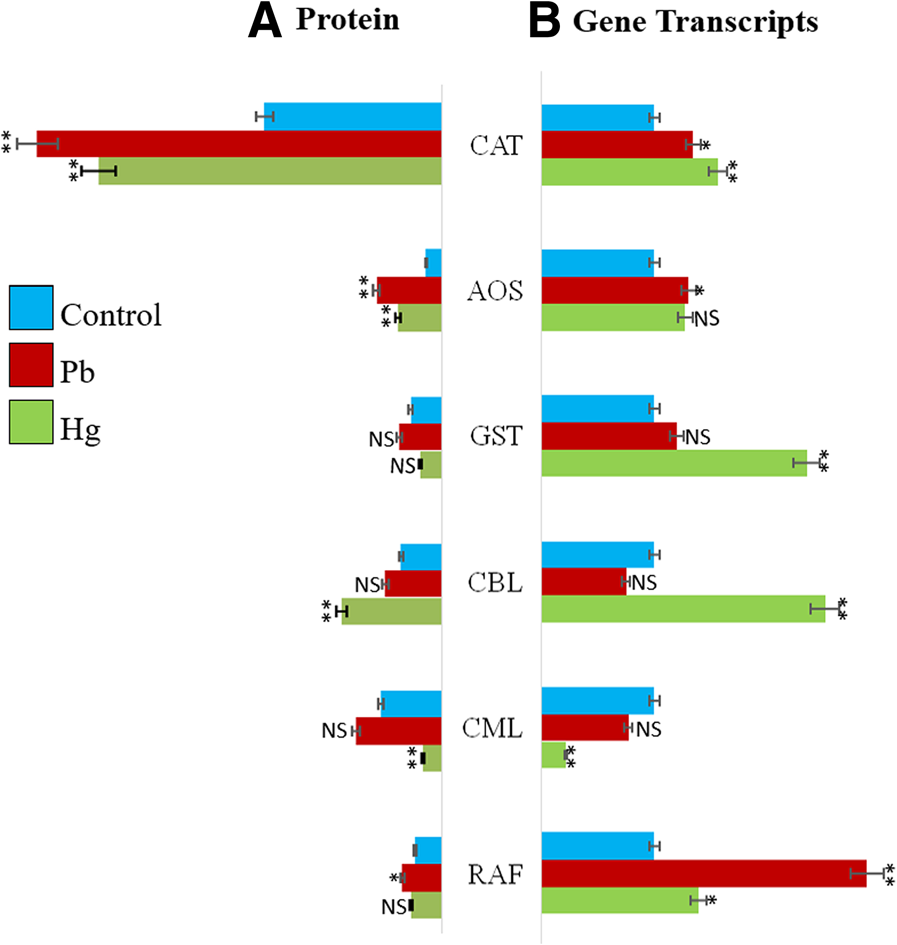
**a-b**. A comparative account of Pb- and Hg-induced changes in relative abundance of some important proteins and related transcripts. The data were normalised against control plants. CAT, catalase; AOS, Allene oxide synthase; GST, Gluitathione S-Transferase; CBL, Calcineurin B like; CML, Calmodulin like; RAF, Rapid alkalinisation factor. All data are presented as the mean ± standard error (SE). The measurements were made using three replicates (*n* = 3). The statistical analyses were carried out using one-way analysis of variance (ANOVA) and means were compare using Tukey’s test. NS = Non-significant, *P ≤ 0.05 and **P ≤ 0.01

Real time quantitative estimation of gene transcripts was achieved and compared to protein expression level (Fig. 12 a-b). A relation between relative abundance of protein (Fig. 12a) and related transcripts (Fig. 12b) reveals a comparative account of protein and related transcript population. Results showed that expression of CAT transcripts was elevated by both Pb and Hg, more under Hg stress. Both Pb and Hg could increase the expression of AOS up to a same level. Levels of GST transcripts were high under Hg stress, compared to a non-significant increase under Pb stress. The abundance of CBL transcripts was also very high under Hg stress whereas some decrease was noted under Pb stress. Abundance of CML was decreased by both stressors. Abundance of RAF transcripts was much higher under Pb stress compared to lower elevation under Hg stress.

## Discussion

Abiotic stresses are denoted by an array of different stress-causing mechanism. Such mechanisms vary from plasmolysis to oxidative stress to interruption in electron flow to biomolecules, cellular degradation/dysfunction, and much more. In turn, plant responds by modulating its transcriptome, proteome and metabolome for defence and repair process, and amelioration of growth and development [21].

Soybean belongs to the legume family which is characterized by N_2_-fixing root nodules that harbour symbiotic bacteria and fixes atmospheric nitrogen to available nitrogenous forms [22]. Thus, their nodules, besides roots, are the primary sites at the risk of metal toxicity. In fact, rate of germination and efficiency of bacteria for nodulation might also be hugely compromised. Therefore, the present study focuses on understanding Pb and Hg induced changes in proteome, selective transcripts and antioxidant enzymes of nitrogen-fixing root nodules in soybean.

### Nodule anatomy was modulated by Pb and hg

Toxic metal induced oxidative stress causes damage to cellular structures and alteration in functions which might be responsible for damage to nodule structures. Both Pb and Hg had differential damaging pattern to the nodule anatomy. It could be due to the differential mechanism of Pb and Hg toxicity; Pb affected the nodule anatomy more whereas Hg affected the bacteroides.

### Both Pb and hg induces oxidative stress in the nodules and free radicals in the leaves

Induction of oxidative stress by toxic metals is a common phenomenon in plant cells [23]. The marker of oxidative stress in both nodules and leaves confirms that magnitude of oxidative damage was higher by Pb than Hg. Perhaps, Pb involves in production of reactive oxygen species more efficiently than Hg.

### Pb induces higher production of hydrogen peroxide and superoxide radicals in leaves

Both Pb and Hg are known to induce oxidative stress [15] in leaves of crop plants. Histochemical localization reveals that Pb produces H_2_O_2_ more efficiently. Hg was not found to elevate the levels of H_2_O_2_. Superoxides were elevated by both Pb and Hg.

### Pb and hg induced changes in protein metabolism

**Fig. 13:**
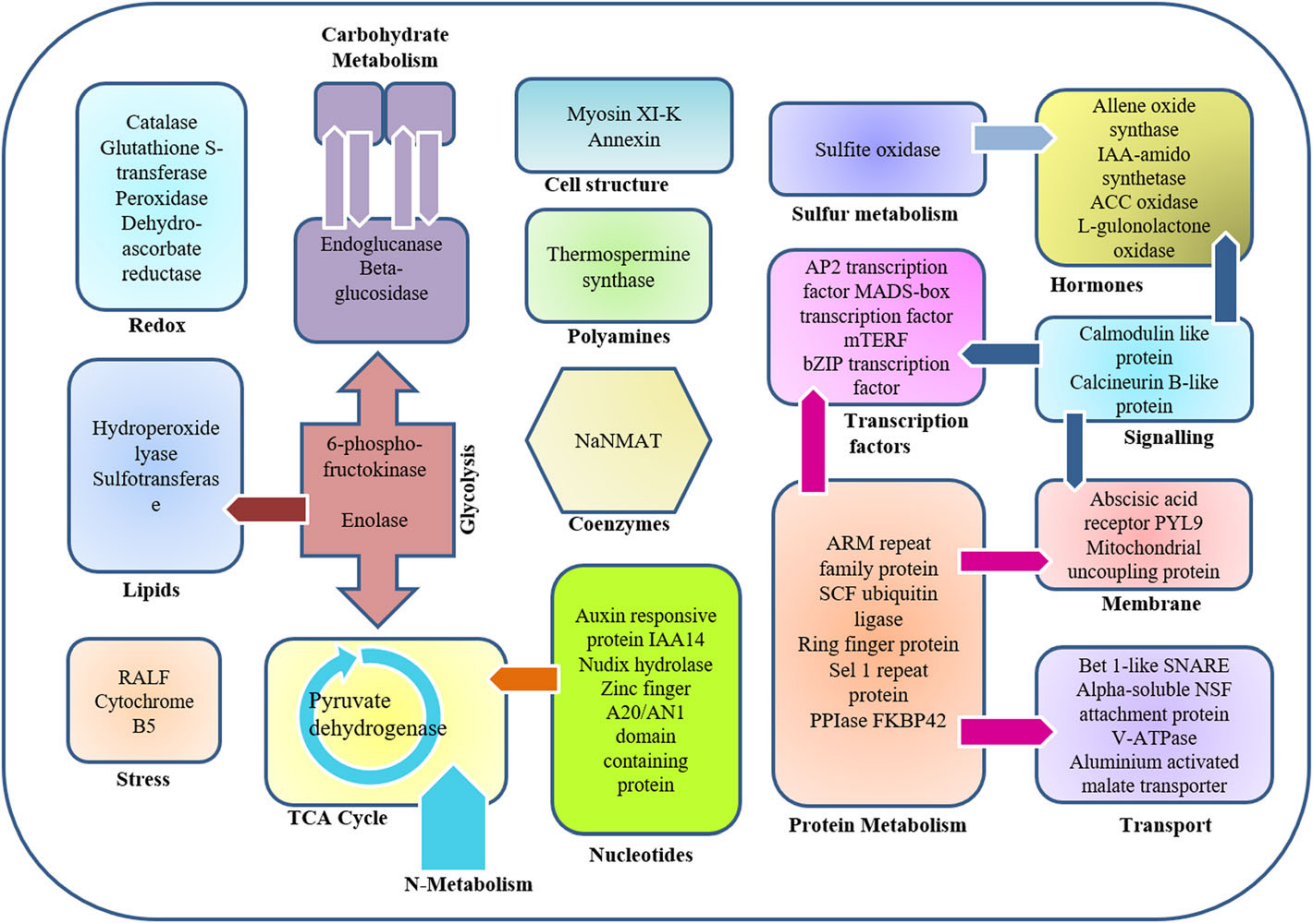
An overview of different pathways with Pb- and Hg-induced changes in abundance of identified proteins

Toxic metals such as Pb and Hg are non-essential and among common contaminants found in polluted soils and water near industrial sites [24]. Plants respond to toxic effects of metals by altered expression of stress associated genes and proteins leading to activation of signalling networks for stress regulation [25]. Protein metabolism related proteins with altered expression due to Pb and Hg stress were identified in our study. These proteins were found to be involved in varied biological processes such as protein glycosylation, folding, ubiquitination, defence response, metal ion binding, transport, and protein synthesis (Fig. 13).

Ubiquitin mediated protein degradation is an important cellular defence response process against environmental stresses. In our study Armadillo/beta-catenin repeat family protein and SCF ubiquitin ligase, SKP1 component were upregulated in both Pb and Hg treatment. Armadillo/beta-catenin repeat family protein belong to E3 ubiquitin ligases which functions as signalling molecule through protein-protein interaction [26]. Abscisic acid (ABA) signalling functions in various environmental perturbances. RING finger protein 141 is positively correlated with ABA signalling and its abundance was down regulated in Pb treatment while upregulated in Hg treatment. Sel1 repeat protein was down regulated in both Pb and Hg treatment. This protein is involved in ER-associated protein degradation for efficient removal of mis-folded proteins. It has been reported that stressed ER aggravate defect in ER-associated protein degradation [27].

Plants respond to environmental changes by acclimatization at various levels including cell turgor, volume, membrane surface modifications and cell expansion which are restricted within cell wall [28]. Membrane trafficking is an integral part of cell growth and development which involves specialized proteins for vesicle traffic and membrane transport. Abundance of ER-Golgi transport protein bet 1-like SNARE was found to be upregulated under Pb and Hg stress while alpha-soluble NSF attachment protein was downregulated in both stresses. FKBPs (FK506-binding proteins) are functionally diverse proteins that belong to Peptidyl-prolyl cis-trans isomerases (PPIases) involved in protein folding and a subset of these proteins are stress responsive [29]. PPIase FKBP42 was found to be upregulated under Pb stress and downregulated under Hg stress.

### Proteins involved in oxidative stress and redox homeostasis

Metal toxicity lead to excessive production of reactive oxygen species (ROS) which cause damage to DNA, proteins, carbohydrates, and lipids resulting in oxidative stress. In plants accumulation of ROS such as hydrogen peroxide and superoxide radicals are indicators of oxidative stress. Response to oxidative stress include changes in gene expression related to growth, development, defence, signalling, and programme cell death (PCD) [30]. In our study, we have identified 18% proteins related to oxidative stress and redox homeostasis. Catalase (CAT), glutathione S-transferase (GST) and peroxidase are involved in detoxification of metal toxicity caused due to oxidative stress [31]. Balestrasse et al. (2008) [32] reported overexpression of CAT gene in nodules of soybean plants exposed to cadmium. In our study, CAT3 was upregulated in both stresses while GST and peroxidase were upregulated in Pb and downregulated in Hg treatment suggesting a pronounced effect of Hg over Pb. Changes in gene expression due to oxidative stress are regulated by network of regulatory transcription factors. AP2 class of transcription factors are known to involve in abiotic stress response [33]. This transcription factor was upregulated in both Pb and Hg treatment suggesting alteration in gene expression due to metal stress.

Small peptides act as signalling molecules in plant growth, development and defence response. One such peptide, rapid alkalinization factor (RAF) which induces extracellular pH elevation was overexpressed in both stresses. Alkalinization of extracellular media in plant helps in functioning of various bioactive peptides which are involved in defence response. RALF was found to arrest root growth in tomato and *Arabidopsis* seedlings and regulates nodulation in *Medicago truncatula* [34, 35]. Oxidative stress causes membrane damage due to lipid peroxidation. Overexpression of Cytochrome b5 in Pb stressed nodules indicate possible membrane repair process as this protein is involved in fatty acid synthesis of ER membrane [36]; however this protein is downregulated in Hg stress.

Dehydroascorbate reductase (DHAR) maintains reduced ascorbate levels in the cells which act as ROS scavenger and detoxify H_2_O_2_ via ascorbate glutathione cycle. Both Pb and Hg stress reduces expression of DHAR. Loscos et al. [37] reported post-translational inhibition of DHAR in nodules of bean plants exposed to stress signalling compound jasmonic acid. Inhibition of DHAR causes apoplast oxidation which is perceived as a stress signal by nodules.

### Nucleotide metabolism related proteins

Nucleotide metabolism is a fundamental process for plant growth and development. Metal stress can affect nucleotide metabolism. ROS mediated damage to different cellular molecules can trigger defence and repair processes involving antioxidant system, growth and cell division which require nucleotide synthesis.

Pb and Hg influenced the abundance of various proteins involved in nucleotide metabolism. Auxin responsive protein IAA14 involved in auxin signalling pathway and which controls lateral root morphogenesis was down regulated by both Pb and Hg stress. Lateral root formation can be suppressed by inhibition of auxin transport [38]. MADS-box transcription factors play important role in plant development. Around 50 MADS-box genes are known to express in *Arabidopsis* roots with most of them having unknown function. *ANR1* is a member of MADS-box family which control lateral root development and respond to nitrogen availability [39]. Contrary to IAA14 expression under Pb and Hg stress, MADS-box transcription factor was upregulated under both the stressors implying positive regulation towards stress response. *Arabidopsis* mitochondrial transcription termination factor family protein (mTERF) encoded by *shot1* (suppressor of hot 1–4) which is a suppressor of heat shock protein HSP101 (*hot 1–4*) provides thermo-tolerance and protection against oxidative damage through altered expression of redox related genes [40].

In the present study, mTERF was up regulated under both Pb and Hg stress indicating possible role in protection against oxidative stress. Nudix hydrolases are enzymes that catalyze hydrolysis of various nucleoside diphosphate derivatives. These enzymes protect against mutagenesis owing to excessive ROS production [41]; [42]. Mitochondrial nudix hydrolase 18 was upregulated by Pb and downregulated by Hg stress. Zinc-finger proteins have diverse biological functions such as binding to DNA, RNA, lipids, and proteins as well as providing acclimation to various biotic and abiotc stresses. In *Arabidopsis* 14 genes encoding stress associated proteins (SAP) having A20/AN1 zinc-finger domains were identified and were grouped based on their domain structure [43].

In this study, we have identified zinc-finger A20 and AN1 domain containing stress associated protein 1 and ADP-ribosylation factor GTPase-activating protein having C4 type zinc finger domain. These proteins were up regulated by Pb and down regulated by Hg stress.

### Carbohydrate metabolism related proteins

Carbohydrates serve as structural components, energy source for plant cells and maintain enzyme activities during stress. In our study, we have identified five proteins related to carbohydrate metabolism with altered expression due to Pb and Hg stress. Enzymes of glycolytic pathway pyruvate dehydrogenase E1 [44] component subunit beta and ATP-dependent 6-phosphofructokinase 3 isoform X2 were up regulated by Pb and down regulated by Hg stress while plastid enolase [45] was downregulated in both stresses. Endoglucanase 19 catalyzes cell wall organization through carbohydrate binding and cellulase activity. *Arabidopsis* beta -glucosidase 15 (BGLU15) degrades flavonol 3-O-beta-glucoside-7-O-alpha-rhamnosides which is a major flavonol that accumulate during abiotic stresses mainly during nitrogen deficiency and low temperature [46]. The expression of both endoglucanase 19 and beta-glucosidase 15 was up regulated by Pb and down regulated by Hg stress.

### Proteins related to hormone and lipid metabolism

Hormones play a crucial role in abiotic stress tolerance in plants through growth and development. Optimum hormone levels are critical for proper physiological functioning of plant cells [47]. In our study Pb and Hg induced prominent changes in expression of proteins related to hormone metabolism. Jasmonates are lipid hormones which functions as signalling molecules in many physiological processes of plants including response to abiotic stress. Allene oxide synthase (AOS) catalyzes the first committed step of jasmonic acid biosynthesis pathway by conversion of 13-hydroperoxylinolenic acid to allene oxide. Germinating soybean seeds release jasmonic acid in rhizosphere which is perceived by *Bradyrhizobium japonicum* for induction of nodulation (*nod)* genes [48]. High abundance of AOS under Pb and Hg stress indicates possible activation of jasmonic acid pathway for signalling and stress response.

Several studies demonstrated the role of auxins in stress response. The active form of IAA is converted to inactive form by conjugation with amino acids which is catalysed by IAA-amido synthetases belonging to group II GH3 protein family. GH3 genes have been shown to participate in biotic and abiotic stress resistance. The *OsGH3–8, OsGH3–1* and *OsGH3–13* genes belonging to group II GH3 family of rice have roles in development and stress resistance [49]. In our study, abundance of IAA-amido synthetase was increased by both stresses. Transcription factors have DNA binding domains which bind at *cis*-elements in promoter regions of abiotic stress responsive genes and induce their expression [50].

Plant basic leucine zipper (bZIP) transcription factors are involved in different growth and developmental processes including abiotic stress signalling. In *Arabidopsis* ER stress associated unfolded protein response (UPR) activates *bZIP28,* which interacts with CCAAT box binding factors NY-Fs for upregulation of ER-stress responsive genes [51]. Transgenic *Arabidopsis* over expressing soybean bZIP genes *GmbZIP132*, *GmbZIP44*, *GmbZIP62*, *GmbZIP78* negatively regulate ABA signalling and confers stress tolerance [52].

Transcription factor TGA6 is the member of bZIP family which is expressed in emerging young roots and is involved in cellular defence against biotic and abiotic stresses. TGA6 regulate gene expression in response to bioactive compounds oxylipins under stressed conditions and *GST25* activity in *Arabidopsis* [53]. In this study bZIP transcription factors were overexpressed in Pb and underexpressed in Hg stress. Role of ethylene in plant defence and abiotic stress tolerance is evident by alteration in expression of stress associated genes in *Arabidopsis*, *Medicago truncatula* and rice exposed to various abiotic stress conditions. 1-aminocyclopropane-1-carboxylate (ACC) oxidase catalyzes conversion of ACC to ethylene. Altered expression of ACC is known to regulate abiotic stress tolerance in *Arabidopsis* and rice [54]. Pb and Hg stress caused downregulation of ACC oxidase in our study which might be due to decreasing level of ethylene.

Lipids are integral part of cell membranes, impart energy for metabolism, maintain growth, development, and stress response [55]. Activation of oxylipin pathway by environmental or developmental stimuli induces synthesis of bioactive compounds called oxylipins. The oxylipin pathway has branch pathways for hydroperoxide lyase (HPL) and allene oxide synthase (AOS) which belong to cytochrome P450s and are stress responsive. HPL and AOS metabolize hydroperoxide fatty acid to different oxylipins [56].

In this study, Pb and Hg treatment caused over expression of hydroperoxide lyase which suggests overproduction of bioactive oxylipins for stress tolerance. Under Pb stress, a newly induced protein sulfotransferase was identified. Activity of several bioactive compounds such as steroids, brassinosteroids, and flavonols is controlled by sulfotransferases which catalyse sulfate group transfer from 3’-Phosphoadenosine-5′-phosphosulfate (PAPS) to hydroxyl or amine group of substrates. Several functional genomics studies of sulfotransferases in *Arabidopsis* characterize various sulfotransferase genes which are regulated by growth, development, and stress response [57].

### Signalling and transport related proteins

The elevation in cytosolic calcium (Ca^2+^) levels is the first signal sensing response towards most environmental stresses. Higher plants have several calcium sensor protein families. Calmodulins (CaM), calmodulin like proteins (CML) and calcineurin B-like (CBL) proteins are well known calcium sensors having multiple calcium binding domains and are involved in abiotic stress response. *AtCML43* is a root tip specific calcium sensor in *Arabidopsis* which was overexpressed when exposed to defence hormone salicylic acid (SA) [58]. In this study, treatment of Pb increased the abundance of CML43 and CML36 whereas Hg decreased the abundance of both proteins. In contrast, calcineurin B-like protein was found to be decreased by Pb and increased by Hg treatment. Calcineurin B-like protein is known to regulate abscisic acid responses in *Arabidopsis* [59]. Abscisic acid receptor PYL9 provide drought tolerance and regulates lateral root formation in *Arabidopsis* [60, 61]. Abundance of ABA PYL9 receptor protein was upregulated by Pb and downregulated by Hg stress.

Transport across membranes is a vital process for maintaining cell homeostasis. Vacuoles maintain cell turgor pressure by osmotic entry of water through V-ATPases which are multisubunit proton pumps localized in plant cell endomembranes. Under environmental stress, maintenance of V-ATPase activity is indispensable for cell survival [62]. Over expression of V-ATPase like protein under Pb and Hg stress suggests increased active transport across membranes for maintenance of cell homeostasis under toxic metal stress.

ROS and lipid peroxidation products can stimulate mitochondrial uncoupling protein(UCP) activity for uncoupling of mitochondria which causes reduction in electron transport chain for limiting ROS formation inside mitochondria. Over-expression of *AtUCP1* gene from *Arabidopsis* confers multiple abiotic stress tolerance in transgenic tobacco plants [63]. The abundance of mitochondrial uncoupling protein 2 was increased in Pb stress and decreased in Hg stress. Aluminium activated malate transporter (ALMT) is a ligand gated anion transporter protein which exudate malate and other inorganic anions for tolerance against Al^3+^ and functions in malate homeostasis and inorganic anion transport in guard cells [64]. ALMT9 protein was overexpressed in both the stresses in our study.

### Miscellaneous proteins

Proteins with diverse cellular processes such as co-factor, vitamin, polyamine metabolism, sulfur assimilation and structural proteins were identified in our study. Nicotinamide/nicotinic acid mononucleotide adenylyltransferase (NaMNAT) is involved in biosynthesis of nicotinamide adenine dinucleotide (NAD) which is a co-factor that regulates cellular redox reactions and ROS signalling in plant development and stress response [65]. NaMNAT was overexpressed in Pb and underexpressed in Hg treatment. L-gulonolactone oxidase (GLOase) is the final precursor of ascorbic acid biosynthesis via *myo*-inositol pathway. Overexpression of GLOase gene in transgenic potato confers increased ascorbate accumulation and provides abiotic stress tolerance [66]. GLOase was overexpressed in both Pb and Hg stress suggesting enhanced accumulation of ascorbate to cope with metal induced oxidative stress.

Myosins are motor proteins involved in cell dynamics. Myosin XI-K is the member of myosin XI family which regulate root hair elongation and organelle trafficking [67]. Downregulation of myosin XI-K under both Pb and Hg stress indicates possible loss of cell motility. Annexins are well documented for their role in environmental stress tolerance. *Arabidopsis* annexin ATANN1 regulates calcium mediated ROS signalling in root epidermal cells under salinity stress [68]. In our study, Annexin D8 was upregulated by Pb and downregulated by Hg stress.

Polyamines in association with brassinosteroids activate ABA and IAA pathways for tolerance against metal stress. Putrescine, spermine, spermidine and thermospermine are common polyamines found in plants. *Arabidopsis ACAULIS5 (ACL5)* gene encodes thermospermine synthase which converts spermidine to thermospermine [69]. In this study, abundance of thermospermine synthase ACAULIS5-like protein was up regulated by Pb and down regulated by Hg stress.

Sulfite oxidase (SOX) is an important enzyme of sulfur assimilation pathway which catalyses re-oxidation of sulfite to sulfate. *SOX* gene in *Arabidopsis* was differentially expressed under various abiotc stress conditions [70]. Abundance of SOX expression was found to be upregulated by Pb and downregulated by Hg stress in root nodules of soybean. Therefore, it was quite evident that an array of proteomic response including numerous metabolic pathways were modulated under metal stress (Fig. 13).

### Correlation of gene transcription with protein expression

Expression pattern of selective key proteins due to Pb and Hg stress was further validated by transcript abundance in Pb and Hg exposed nodules using qPCR analysis. These genes included those that encode CAT, AOS, GST, CBL, CML and RAF. Expression of CAT transcripts was elevated by both Pb and Hg, more under Hg stress. Both Pb and Hg could increase the expression of AOS up to the same level indicating the possible role in defence. However, levels of GST transcripts were high under Hg stress, compared to a non-significant increase under Pb stress. The data indicates towards a special feature of plant to increase the level of GST. The levels of CBL transcripts were also high under Hg stress whereas some decrease was noted under Pb stress, indicating its role during metal stress. Expression of CML was decreased under both stressors and hence seems a major protein adversely affected by metal stress. Interestingly, quantity of RAF transcripts was much higher under Pb stress compared to lower elevation under Hg stress. The data clearly indicatethe important role of RAF under Pb stress.

Post mRNA synthesis, many regulatory processes such as post transcriptional, translational and protein degradation control protein abundances. Proteins have longer half-life as compared to mRNA [71]. The rate of mRNA transcription is much lower than protein translation. The biochemical diversity of proteins can cause variation in protein and mRNA correlation level which might be the cause of difference in expression pattern of GST, CBL and CML genes as compared to protein expression.

## Conclusions

Our study provides novel insights into proteomic and ecophysiological responses to Pb and Hg induced phytotoxicity in nodules of soybean plant. Symbiotic nodules are among the primary sites that encounter and counter metal stress. For strengthening the defence, understanding the response of nodule becomes pivotal in order to design proper strategies. In our study, it is evident that toxic metals (Pb and Hg) cause differential expression of proteins and transcript abundance in soybean nodules even when present in low concentrations. Nodules responded in an array of responses at the level of cellular antioxidants, transcription regulation, modulation of protein profile and causes prominent changes in nodule anatomy and bacteroidal volume. There is an increased abundance in defence, repair and development related proteins due to Pb and Hg stress. Pb newly induced a unique protein, sulfotransferase which controls the activity of several bioactive compounds involved in stress response. Correlation between protein expression and gene transcript levels due to Pb and Hg stress provided clues about regulation at transcriptional level. Pb and Hg stress responses in soybean root nodules has increased our knowledge regarding the mechanisms of stress tolerance which can be helpful in development of stress tolerant varieties through gene transfer and marker-assisted breeding. Such study would contribute not only to fill the gap of our understanding about nodule but also raise a hope of improved yield and production of soybean.

## Additional files

Additional file 1: **Table S1.** Program with varied current ramp, voltage, duration and total current used to perform for isoelectric focusing of protein extracted from root nodules of *Glycine max* L. Merr. (DOCX 13 kb)

Additional file 2: **Table S2.** Summary of genes selected for qRT-PCR analysis with their corresponding forward and reverse primers and their attributed including melting temperature (T_m_), GC content (%) and product size. (DOCX 14 kb)

Additional file 3: **Table S3.** Information about conditions used in quantification assay of qRT-PCR (DOCX 13 kb)

Additional file 4: **Table S4.** Information about qRT-PCR run conditions (DOCX 12 kb)

Additional file 5: **Figure S1.** Impact of Pb and Hg stress on some key proteins of *Glycine max* L. Changes in actual relative abundance, bar graphs and picture have been demonstrated. (PPTX 1266 kb)

Additional file 6: **Table S5.** List of proteins identified through MALDI TOF MS/MS and their theoretical/practical molecular weight and *pI* values, percent sequence coverage, NCBI accession, SoyKB gene ID’s, biological function, and cellular component. (DOCX 25 kb)

## Abbreviations

2DE: 2-Dimensional Electrophoresis
ABA: Abscisic Acid
ACN: Acetrinitrile
ALMT: Aluminium activated malate transporter
ANOVA: Analysis of variance
AOS: Allene oxide synthase
APX: Ascorbate peroxidase
As: Arsenic
ATSDR: Agency for Toxic Substances and Disease Registry
BSA: Bovine serum albumin
CAT: Catalase
CBL: Calcineurin B like
Cd: Cadmium
CML: Calmodulin like
CPCB: Central Pollution Control Board
CT: Cycle threshold
DTT: Dithiothreitol
EDTA: Ethylenediaminetetraacetic acid
FAOSTAT: Food and agriculture organization corporate statistical database
FICCI: *Federation of Indian Chambers of Commerce and Industry*
GST: Glutathione S-transferase
H_2_O_2_: Hydrogen peroxide
Hg: Mercury
HgCl_2_: Mercuric chloride
HNE: 4-hydroxy-2-nonenal
IAA: Indole-3-acetic acid
IAA: Iodoacetamide
IEF: Iso Electric Focusing
IPG: Immobilized pH Gradient
MALDI TOF MS: Matrix-assisted laser desorption/ionization- time-of-flight mass spectrometry
MDA: Malondialdehyde
MP: Madhya Pradesh
MS: Mass spectrometry
NADPH: Nicitinamide adeninedinucleotide phosphate
NH_4_HCO_3_: Ammonium bicarbonate
Pb: Lead
PbCl_2_: Lead chloride
PCD: Programme cell death
PCR: Polymerase chain reaction
PEP: phosphoenolpyruvate
pI: Isoelectric point
PLS-DA: Partial least squares discriminant analysis
PUFAs: Polyunsaturated fatty acids
qRT-PCR: Quantitative real time polymerase chain reaction
RAF: Rapid alkalinisation factor
ROS: Reactive oxygen species
RT-PCR: Reverse transcription polymerase chain reaction
SDS: Sodium Dodecyl Sulfate
SDS-PAGE: Sodium dodecyl sulfate polyacrylamide gel electrophoresis
TBARS: Thiobarbituric acid reactive substances
TFA: Trifluoroacetic acid
UCPs: Mitochondrial uncoupling proteins
USDA: United States Department of Agriculture
VIP: Variable importance in projection
YEM: Yeast Extract-Mannitol
