# Supplementary material for: Proteomic and ecophysiological responses of soybean (Glycine max L.) root nodules to Pb and hg stress
Source: BMC Plant Biol. 2018 Nov 14;18:283. doi: 10.1186/s12870-018-1499-7 (PMC6237034; doi:10.1186/s12870-018-1499-7)
Supplement: Supplementary file 1 — Table S1. Program with varied current ramp, voltage, duration and total current used to perform for isoelectric focusing of protein extracted from root nodules of Glycine max L. Merr. (DOCX 13 kb) [file 12870_2018_1499_MOESM1_ESM.docx]

**Supplementary Table S1:**

Program with varied current ramp, voltage, duration and total current used to perform for isoelectric focusing of protein extracted from root nodules of *Glycine max* L. Merr.

| Step no. | Current Ramp mode | Volts  (V) | Duration  (h) | Total Current (Vh) |
| --- | --- | --- | --- | --- |
| 1  2  3  4  5  6 | Rapid  Rapid  Rapid  Rapid  Linear  Slow | 250  500  1000  2000  3866.67  500 | 1.0  1.0  2.0  2.0  15.0  0.5 | 250  500  2000  4000  58000  250 |
| Total | | | | 65000 |
