# Supplementary material for: Proteomic and ecophysiological responses of soybean (Glycine max L.) root nodules to Pb and hg stress
Source: BMC Plant Biol. 2018 Nov 14;18:283. doi: 10.1186/s12870-018-1499-7 (PMC6237034; doi:10.1186/s12870-018-1499-7)
Supplement: Supplementary file 2 — Table S2. Summary of genes selected for qRT-PCR analysis with their corresponding forward and reverse primers and their attributed including melting temperature (Tm), GC content (%) and product size. (DOCX 14 kb) [file 12870_2018_1499_MOESM2_ESM.docx]

**Supplementary Table S2:**

Summary of genes selected for qRT-PCR analysis with their corresponding forward and reverse primers and their attributed including melting temperature (T_m_), GC content (%) and product size.

| **Genes and Primers** | **T_m_ (^o^C)** | **GC %** | | **Product Size (bp)** |
| --- | --- | --- | --- | --- |
| 1. Catalase (CAT) |  |  | |  |
| Forward Primer: **CACGTGTCACCCATGAAATC** | 59.8 | 50 | | 70 |
| Reverse Primer: **TGTCCAAGAGAACGATCAGC** | 59 | 50 | |  |
| 1. Allene Oxide Synthase (AOS) | | | | |
| Forward Primer: **AGTTCTTCAAGTCCCGCATC** | 59.3 | | 50 | 115 |
| Reverse Primer: **GTTTTTGCGTCGAGGAGAAC** | 59.9 | | 50 |  |
| 1. Glutathione S-Transferase (GST) | | | | |
| Forward Primer: **CCTGCCAAGAAAGAGTTTGG** | 59.8 | | 50 | 105 |
| Reverse Primer: **AGGACCGGCTTGATGTATTG** | 60 | | 50 |  |
| 1. CalcineurinB Like (CBL) | | | | |
| Forward Primer: **TAATCTTGCGGCAGAGACAG** | 59.2 | | 50 | 104 |
| Reverse Primer: **TGCTTATCAGTCCGTCATCG** | 59.8 | | 50 |  |
| 1. Calmodulin Like (CML) | | | | |
| Forward Primer: **CTCAAACTAAGCCCCAAACG** | 59.7 | | 50 | 116 |
| Reverse Primer: **TGATTTGCGGTGGAAGAGTC** | 61.2 | | 50 |  |
| 1. Rapid Alkalinization Factor (RAF) | | | | |
| Forward Primer: **GACAACAACACCATGCCAAG** | 60 | | 50 | 101 |
| Reverse Primer: **ACTGCGAGGTTGCTAGAATG** | 58.1 | | 50 |  |
| 1. SOY ACTIN | | | | |
| Forward Primer: **TGCTGTGTGTGTTGCTGTT** | 58.4 | | 47.4 | 141 |
| Reverse Primer: **AAGTGCATGTCTGTCGATGA** | 57.3 | | 45 |  |
