# Supplementary material for: Proteomic and ecophysiological responses of soybean (Glycine max L.) root nodules to Pb and hg stress
Source: BMC Plant Biol. 2018 Nov 14;18:283. doi: 10.1186/s12870-018-1499-7 (PMC6237034; doi:10.1186/s12870-018-1499-7)
Supplement: Supplementary file 3 — Table S3. Information about conditions used in quantification assay of qRT-PCR (DOCX 13 kb) [file 12870_2018_1499_MOESM3_ESM.docx]

#### Supplementary Table S3: Information about conditions used in quantification assay of qRT-PCR

| **Attribute** | **Remark** |
| --- | --- |
| Threshold | 0.05111 |
| Left Threshold | 1.000 |
| Standard Curve Imported | No |
| Standard Curve (1) | N/A |
| Standard Curve (2) | N/A |
| Start normalising from cycle | 1 |
| Noise Slope Correction | Yes |
| No Template Control Threshold | 10% |
| Reaction Efficiency Threshold | Disabled |
| Normalisation Method | Dynamic Tube Normalisation |
| Digital Filter | Light |
| Sample Page | Page 1 |
| Imported Analysis Settings |  |
