# Supplementary material for: Proteomic and ecophysiological responses of soybean (Glycine max L.) root nodules to Pb and hg stress
Source: BMC Plant Biol. 2018 Nov 14;18:283. doi: 10.1186/s12870-018-1499-7 (PMC6237034; doi:10.1186/s12870-018-1499-7)
Supplement: Supplementary file 4 — Table S4. Information about qRT-PCR run conditions (DOCX 12 kb) [file 12870_2018_1499_MOESM4_ESM.docx]

**Supplementary Table S4:** Information about qRT-PCR run conditions

| **Cycle** | **Cycle Point** |
| --- | --- |
| Hold @ 95°C, 10 min 0 secs |  |
| Cycling (45 repeats) | Step 1 @ 95 °C, hold 15 secs |
|  | Step 2 @ 55 °C, hold 20 secs |
|  | Step 3 @ 72 °C, hold 20 secs, acquiring to Cycling A([Green][1][1]) |
