# Supplementary material for: Proteomic and ecophysiological responses of soybean (Glycine max L.) root nodules to Pb and hg stress
Source: BMC Plant Biol. 2018 Nov 14;18:283. doi: 10.1186/s12870-018-1499-7 (PMC6237034; doi:10.1186/s12870-018-1499-7)
Supplement: Supplementary file 5 — Figure S1. Impact of Pb and Hg stress on some key proteins of Glycine max L. Changes in actual relative abundance, bar graphs and picture have been demonstrated. (PPTX 1266 kb) [file 12870_2018_1499_MOESM5_ESM.pptx]

## Slide 1
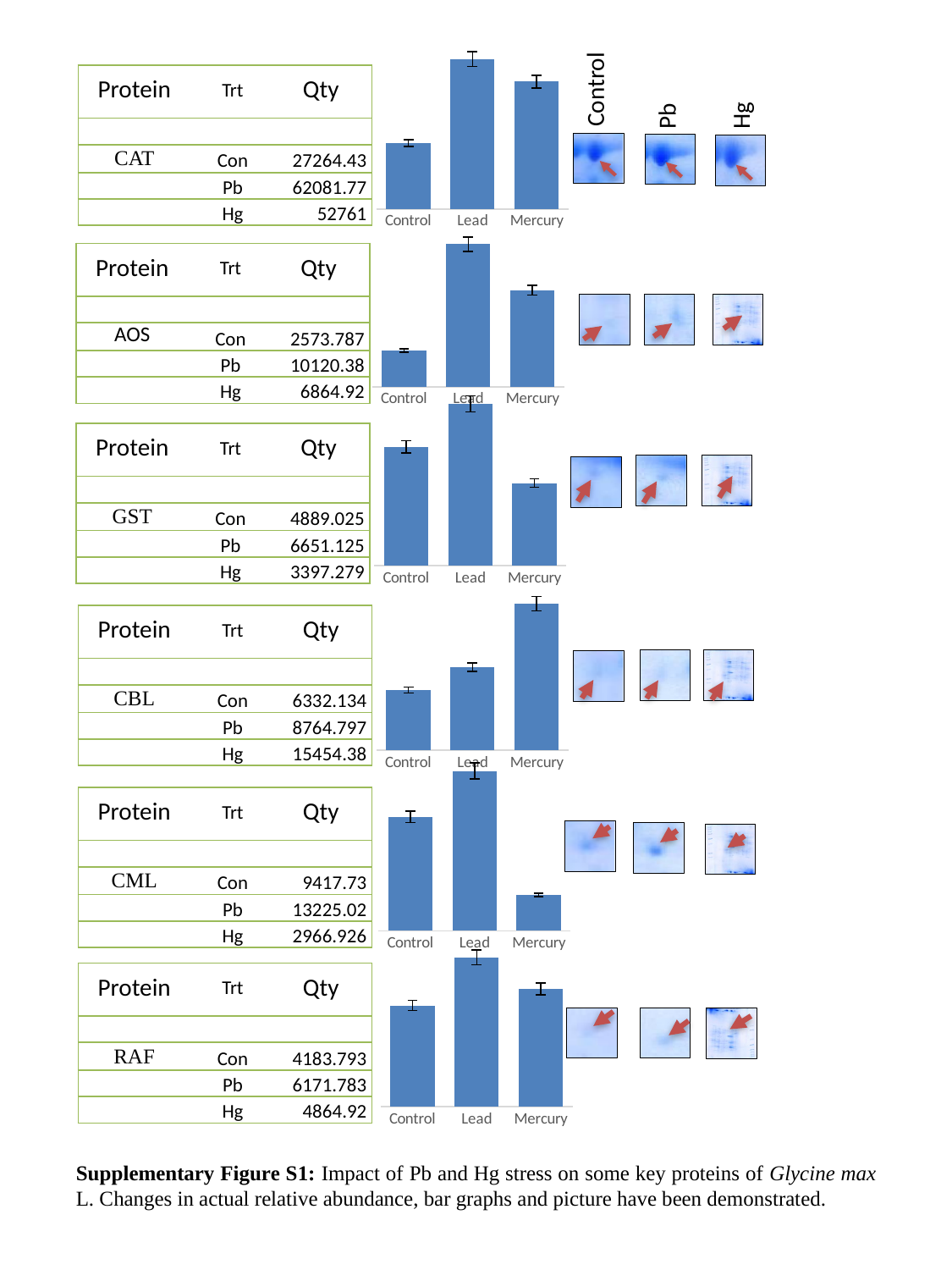

### Chart
| Category | |
|---|---|
| Control | 27264.4336 |
| Lead | 62081.7656 |
| Mercury | 52760.970700000005 || Protein | Trt | Qty | |
| --- | --- | --- | --- |
| | | | |
| CAT | Con | 27264.43 | |
| | Pb | 62081.77 | |
| | Hg | 52761 | |
Control
Hg
Pb
### Chart
| Category | |
|---|---|
| Control | 2573.786599999994 |
| Lead | 10120.375999999953 |
| Mercury | 6864.922900000001 || Protein | Trt | Qty | |
| --- | --- | --- | --- |
| | | | |
| AOS | Con | 2573.787 | |
| | Pb | 10120.38 | |
| | Hg | 6864.92 | |
### Chart
| Category | |
|---|---|
| Control | 4889.0249 |
| Lead | 6651.125000000011 |
| Mercury | 3397.2793 || Protein | Trt | Qty | |
| --- | --- | --- | --- |
| | | | |
| GST | Con | 4889.025 | |
| | Pb | 6651.125 | |
| | Hg | 3397.279 | |
### Chart
| Category | |
|---|---|
| Control | 6332.1338000000005 |
| Lead | 8764.796899999983 |
| Mercury | 15454.3838 || Protein | Trt | Qty | |
| --- | --- | --- | --- |
| | | | |
| CBL | Con | 6332.134 | |
| | Pb | 8764.797 | |
| | Hg | 15454.38 | |
### Chart
| Category | |
|---|---|
| Control | 9417.732400000023 |
| Lead | 13225.0225 |
| Mercury | 2966.9263 || Protein | Trt | Qty | |
| --- | --- | --- | --- |
| | | | |
| CML | Con | 9417.73 | |
| | Pb | 13225.02 | |
| | Hg | 2966.926 | |
### Chart
| Category | |
|---|---|
| Control | 4183.792500000001 |
| Lead | 6171.783200000001 |
| Mercury | 4864.922900000001 || Protein | Trt | Qty | |
| --- | --- | --- | --- |
| | | | |
| RAF | Con | 4183.793 | |
| | Pb | 6171.783 | |
| | Hg | 4864.92 | |
Supplementary Figure S1: Impact of Pb and Hg stress on some key proteins of Glycine max L. Changes in actual relative abundance, bar graphs and picture have been demonstrated.
