# Supplementary material for: Proteomic and ecophysiological responses of soybean (Glycine max L.) root nodules to Pb and hg stress
Source: BMC Plant Biol. 2018 Nov 14;18:283. doi: 10.1186/s12870-018-1499-7 (PMC6237034; doi:10.1186/s12870-018-1499-7)
Supplement: Supplementary file 6 — Table S5. List of proteins identified through MALDI TOF MS/MS and their theoretical/practical molecular weight and pI values, percent sequence coverage, NCBI accession, SoyKB gene ID’s, biological function, and cellular component. (DOCX 25 kb) [file 12870_2018_1499_MOESM6_ESM.docx]

**Supplementary Table S5:** List of proteins identified through MALDI TOF MS/MS and their theoretical/practical molecular weight and *pI* values, percent sequence coverage, NCBI accession, SoyKB gene ID’s, biological function, and cellular component.

| Spot No. | Practical Mw/pI | Theoretical Mw/pI | Mascot Score/  %Seq cov. | Homologues  Protein | NCBI Accession | Homolog SoyKB Gene ID | Cellular Component | Biological Process |
| --- | --- | --- | --- | --- | --- | --- | --- | --- |
| 1 | 77.98/5.8 | 78.32/6.33 | 42/23 | *BZIP transcription factor* | XP_003630050 | Glyma05g31400.1 | Nucleus | Stress response, Transcription |
| 2 | 63.7/6.47 | 67.53/6.41 | 53/27 | *Indole-3-acetic acid-amido synthetase GH3.6* | KHN06043 | Glyma06g40860.1 | Cytoplasm | Auxin-activated signaling pathway |
| 3 | 64.18/5.7 | 69.86/5.55 | 42/25 | *AAA ATPase, putative, partial* | XP_013441349 | Glyma19g02180.1 | Membrane, Mitochondrion | Response to abscisic acid/response to salt stress |
| 4 | 63.76/5.23 | 56.96/6.56 | 58/29 | *Catalase 3* | AKP21071 | Glyma14g39810.1 | mitochondrion/ peroxisome | Response to oxidative stress |
| 5 | 64.44/5.16 | 69.45/5.96 | 51/28 | *Aluminum activated malate transporter 9* | XP_010318605 | Glyma05g35190.1 | Vacuole membrane | malate transmembrane transport |
| 6 | 61.35/6.65 | 50.98/8.07 | 48/29 | *Endoglucanase 19* | XP_013735550 | Glyma05g36930.1 | extracellular region | Carbohydrate metabolism, Cell wall biogenesis/degradation |
| 7 | 61.59/6.47 | 65.76/7.79 | 43/23 | *Probable L-gulonolactone oxidase 6* | XP_014498948 | Glyma19g44870.1 | membrane | Ascorbate biosynthesis |
| 8 | 60.32/5.98 | 56.88/6.87 | 47/25 | *Beta-glucosidase 15-like isoform X1* | XP_009143028 | Glyma12g36870.1 | Apoplast, Secreted | Glycosidase, Hydrolase |
| 9 | 60.74/5.82 | 56.57/6.43 | 52/30 | *Hydroperoxide lyase, putative isoform 1* | XP_007049106 | Glyma12g31770.1 | Membrane | Fatty acid metabolic process |
| 10 | 61.43/5.58 | 57.04/7.02 | 57/31 | *Premnaspirodiene oxygenase-like* | XP_010921587 | Glyma07g20430.1 | Membrane | Monooxygenase, Oxidoreductase |
| 11 | 60.71/5.22 | 63.84/5.98 | 49/27 | *Armadillo/betacatenin repeat family protein* | XP_002888089 | Glyma07g30900.1 | intracellular | Uubiquitin-protein transferase activity |
| 12 | 60.72/5.17 | 52.23/6.07 | 48/25 | *Plastid enolase* | ABO36543 | Glyma18g22780.1 | phosphopyruvate hydratase complex | Glycolytic process |
| 13 | 54.96/6.38 | 58.26/8.0 | 47/31 | *Allene oxide synthase* | XP_002510320 | Glyma14g08560.1 | mitochondrion | MAPK cascade/regulation of hydrogen peroxide metabolic process |
| 14 | 54.06/6.1 | 41.74/6.86 | 47/37 | *ATP-dependent 6-phosphofructokinase 3 isoform X2* | XP_014631749 | Glyma06g09320.2 | Cytoplasm | Glycolytic process through fructose-6-phosphate/root epidermal cell differentiation |
| 15 | 54.34/6 | 50.13/6.97 | 45/36 | *beta-1,3-galactosyltransferase 7-like* | XP_008352694 | Glyma14g33700.1 | Golgi apparatus, Membrane | Protein glycosylation |
| 16 | 54.87/5.71 | 47.23/9.69 | 57/40 | *mitochondrial transcription termination factor family protein* | NP_176388 | Glyma18g12810.1 | Mitochondrion | Regulation of transcription, DNA-templated |
| 17 | 53.26/5.37 | 52.89/7.67 | 56/40 | *probable ADP-ribosylation factor GTPase-activating protein AGD6* | XP_014496155 | Glyma19g35620.1 | Cytosol/Nucleus | GTPase activation |
| 18 | 53.22/5.31 | 47.62/8.79 | 50/30 | *myosin XIK partial* | ADV74831 | Glyma20g36970.1 | myosin complex | ATP binding/motor activity |
| 19 | 50.58/5.54 | 58.03/7.22 | 56/33 | *ubiquitin-protein ligase/ zinc ion binding protein* | NP_001148993 | Glyma19g05660.1 | Cytoplasm, Nucleus | Regulation of proteasomal ubiquitin-dependent protein catabolic process |
| 20 | 45.7/6.54 | 38.03/5.90 | 78/53 | *manganese-dependent ADP-ribose/ CDP-alcohol diphosphatase* | XP_009362925 | Glyma06g18600.1 | Cytosol | metal ion binding |
| 21 | 45.38/6.27 | 35.73/8.94 | 49/45 | *Annexin D8* | XP_013452727 | Glyma09g30190.4 | plasmodesma | response to abscisic acid/osmotic stress |
| 22 | 40.34/5.76 | 29.97/6.45 | 43/43 | *Dehydroascorbate reductase* | NP_001275053 | Glyma11g33700.1 | cytoplasm | glutathione metabolic process |
| 23 | 36.97/6.65 | 39.39/9.78 | 54/43 | *Ribosomal RNA small subunit methyltransferase, mitochondrial* | XP_014520158 | Glyma01g37520.1 | Mitochondrion | adenosine metabolic process |
| 24 | 35.68/6.05 | 32.58/6.27 | 58/42 | *Alpha-soluble NSF attachment protein 2 isoform X2* | XP_003534662 | Glyma09g41590.2 | Membrane | Protein transport |
| 25 | 31.93/6.66 | 30.79/5.14 | 42/36 | *Transcription factor TGA5* | XP_008439732 | Glyma05g32880.1 | Nucleus | defense response/salicylic acid mediated signaling pathway |
| 26 | 33.3/6.14 | 33.09/9.55 | 55/47 | *Mitochondrial uncoupling protein 2* | AAU11463 | Glyma03g14780.1 | Mitochondrion inner membrane | Stress response, Transport |
| 27 | 32.33/6.05 | 39.58/5.38 | 52/42 | *Thermospermine synthase ACAULIS5-like* | XP_015083600 | Glyma17g34300.1 | cytoplasm | Polyamine biosynthesis |
| 28 | 31.46/5.4 | 36.55/5.15 | 53/42 | *1-aminocyclopropane-1-carboxylate oxidase* | NP_001234638 | Glyma15g11930.1 | cell wall/cytosol/endoplasmic reticulum/Golgi apparatus/plasma membrane | ethylene biosynthetic process/ Plant defense |
| 29 | 30.67/5.23 | 27.42/6.63 | 56/42 | *Glutathione S-transferase L3-like isoform X1* | XP_009631738 | Glyma19g36080.1 | Cytoplasm | Detoxification |
| 30 | 28.59/5.03 | 17.91/4.70 | 51/69 | *SCF ubiquitin ligase, SKP1 component* | XP_003621659 | Glyma11g08440.1 | integral component of membrane | ubiquitin-dependent protein catabolic process |
| 31 | 27.59/5.02 | 21.59/8.09 | 5963 | *MADS-box transcription factor, partial* | BAT57292 | Glyma06g48270.3 | Nucleus | lateral root development/response to nitrate |
| 32 | 27.02/4.91 | 24.52/4.75 | 54/47 | *Calcineurin B-like protein* | AHA98342 | Glyma05g05580.1 | Cell membrane | Abscisic acid signaling pathway, Stress response |
| 33 | 26.72/6.19 | 31.11/8.46 | 49/38 | *Sel1 repeat protein* | XP_013446324 | Glyma08g37250.2 | Endoplasmic reticulum membrane | ER-associated ubiquitin-dependent protein catabolic process |
| 34 | 26.54/5.79 | 27.46/6.23 | 53/49 | *nicotinamide/nicotinic acid mononucleotide adenylyltransferase-like* | XP_013665778 | Glyma14g01730.2 | Nucleus | NAD biosynthetic process |
| 35 | 25/6.6 | 27.44/8.20 | 47/42 | *Auxin-responsive protein IAA14-like isoform X2* | XP_011030586 | Glyma10g32340.1 | Nucleus | Auxin signaling pathway/lateral root morphogenesis |
| 36 | 24.83/6.11 | 31.39/6.72 | 46/41 | *probable protein phosphatase 2C 10* | XP_003522796 | Glyma04g11000.1 | Membrane | protein serine/threonine phosphatase activity |
| 37 | 25.08/5.91 | 23.00/4.48 | 46/53 | *calmodulin-like protein CML36* | AGV22098 | Glyma03g28650.1 | Cytosol/Nucleus | calcium-mediated signaling/response to abscisic acid/response to salt stress |
| 38 | 24.16/5.66 | 31.41/8.40 | 61/37 | *Peroxidase 41* | KHN37877 | Glyma10g34190.1 | extracellular region | hydrogen peroxide catabolic process/response to oxidative stress |
| 39 | 22.83/5.95 | 15.18/5.43 | 50/53 | *Cytochrome b5 isoform 1* | KYP44880 | Glyma06g13840.3 | Membrane | metal ion binding |
| 40 | 22.41/5.8 | 21.09/6.07 | 46/56 | *abscisic acid receptor PYL9-like* | XP_009104333 | Glyma07g06270.2 | cytoplasm/nucleus/plasma membrane | Abscisic acid signaling pathway |
| 41 | 22.77/5.72 | 18.60/9.10 | 51/49 | *ribosomal RNA small subunit methyltransferase E-like* | XP_014618326 | Glyma20g27210.2 | Cytosol | rRNA processing |
| 42 | 21.03/6.5 | 23.73/9.27 | 47/42 | *nudix hydrolase 18, mitochondrial* | XP_003534133 | Glyma09g30390.1 | Mitochondrion | hydrolase activity/metal ion binding |
| 43 | 18.81/6.11 | 22.61/5.03 | 47/41 | *elongation factor 2-like, partial* | XP_014623559 | Glyma15g40840.1 | Cytoplasm | Protein biosynthesis |
| 44 | 12.17/5.72 | 12.86/4.54 | 50/52 | *AP2 domain class transcription factor* | XP_013468692 | Glyma20g34560.1 | Nucleus | lateral root development/response to osmotic stress/root meristem growth |
| 45 | 11.19/6.08 | 21.27/4.83 | 48/52 | *probable calcium-binding protein CML43* | XP_008393916 | Glyma04g08300.1 | Cytoplasm/Vacuole/Plasma membrane/Nucleus | plant defense response/response to cadmium ion |
| 46 | 10.4/6.84 | 20.74/6.31 | 45/68 | *Sulfotransferase 17* | EMS55393 | Glyma13g26830.1 | Cytoplasm | glucosinolate biosynthetic process |
| 47 | 9.66/6.1 | 8.97/7.71 | 41/41 | *Rapid alkalinization factor* | ACY25867 | Glyma19g39730.1 | extracellular region | Calcium-mediated signaling/Regulation plant stress, growth, and development |
| 48 | 5.71/6.88 | 8.89/5.45 | 57/78 | *bet1-like protein At4g14600 isoform X3* | XP_015583747 | Glyma14g35710.1 | Golgi apparatus membrane/Endoplasmic reticulum membrane | ER-Golgi transport |
| 49 | 5.62/6.07 | 6.41/4.97 | 39/67 | *Pyruvate dehydrogenase E1 component subunit beta, partial* | KVH99736 | Glyma14g02380.2 | Mitochondrion matrix | Glycolysis |
| 50 | 4.79/5.65 | 14.61/8.83 | 44/61 | *zinc finger A20 and AN1 domain-containing stress-associated protein 1-like* | XP_003550893 | Glyma17g14580.1 | Cytosol/Nucleus | Environmental stress response |
| 51 | 5.13/5.27 | 9.47/8.93 | 59/94 | *Vacuolar ATP synthase catalytic subunit-related / V-ATPase-related / vacuolar proton pump-related* | XP_007030175 | Glyma17g04530.1 | vacuolar membrane, vacuole | protein phosphorylation |
| 52 | 5.67/6.82 | 12.22/7.68 | 49/53 | *glutaredoxin-C5-like* | XP_012435721 | Glyma16g05730.1 | Cytoplasm/Nucleus | cell redox homeostasis |
| 53 | 50/6.2 | 44.66/8.80 | 51/33 | *Sulfite oxidase* | KMZ69721 | Glyma19g18330.1 | Peroxisome/Mitochondrion | sulfur compound metabolic process |
| 54 | 44.24/6.02 | 37.72/6.11 | 51/32 | *Peptidyl-prolyl cis-trans isomerase-like 3* | KYP39352 | Glyma18g48350.1 | Nucleus | protein folding |
| 55 | 22.89/5.06 | 28.93/5.68 | 54/26 | *RING finger protein 141* | KHN08537 | Glyma13g31450.1 | Membrane | defense response/protein ubiquitination |
